# Inhibition of CDK1 Overcomes Oxaliplatin Resistance by Regulating ACSL4‐mediated Ferroptosis in Colorectal Cancer

**DOI:** 10.1002/advs.202301088

**Published:** 2023-07-10

**Authors:** Kaixuan Zeng, Weihao Li, Yue Wang, Zifei Zhang, Linjie Zhang, Weili Zhang, Yue Xing, Chi Zhou

**Affiliations:** ^1^ Precision Medical Research Institute the Second Affiliated Hospital of Xi'an Jiaotong University Xi'an 710000 China; ^2^ Department of Colorectal Surgery Sun Yat‐sen University Cancer Center Guangzhou 510060 China; ^3^ State Key Laboratory of Oncology in South China Collaborative Innovation Center for Cancer Medicine Sun Yat‐sen University Cancer Center Guangzhou 510060 China; ^4^ Department of Gastroenterology the First Affiliated Hospital of Nanchang University Nanchang 330006 China; ^5^ IIT Project Management Office the First Affiliated Hospital of Nanchang University Nanchang 330006 China; ^6^ Guangdong Provincial Key Laboratory of Malignant Tumor Epigenetics and Gene Regulation Medical Research Center Sun Yat‐Sen Memorial Hospital Sun Yat‐Sen University Guangzhou 510120 China; ^7^ Breast Tumor Center Sun Yat‐Sen Memorial Hospital Sun Yat‐Sen University Guangzhou 510120 China

**Keywords:** ACSL4, CDK1, colorectal cancer, ferroptosis, oxaliplatin resistance

## Abstract

Oxaliplatin is a widely used chemotherapy drug for patients with advanced colorectal cancer (CRC); however, frequent drug resistance limits its therapeutic efficacy in patients. Here, this work identifies cyclin‐dependent kinase 1 (*CDK1*) as a critical contributor to oxaliplatin resistance via in vitro and in vivo CRISPR/Cas9 screening. *CDK1* is highly expressed in oxaliplatin‐resistant cells and tissues due to the loss of N6‐methyladenosine modification. Genetic and pharmacological blockade of *CDK1* restore the susceptibility of CRC cells to oxaliplatin in vitro and in cell/patient‐derived xenograft models. Mechanistically, CDK1 directly binds to and phosphorylates Acyl‐CoA synthetase long‐chain family 4 (ACSL4) at S447, followed by recruitment of E3 ubiquitin ligase UBR5 and polyubiquitination of ACSL4 at K388, K498, and K690, which leads to ACSL4 protein degradation. Reduced ACSL4 subsequently blocks the biosynthesis of polyunsaturated fatty acid containing lipids, thereby inhibiting lipid peroxidation and ferroptosis, a unique iron‐dependent form of oxidative cell death. Moreover, treatment with a ferroptosis inhibitor nullifies the enhancement of CRC cell sensitivity to oxaliplatin by CDK1 blockade in vitro and in vivo. Collectively, the findings indicate that *CDK1* confers oxaliplatin resistance to cells by suppressing ferroptosis. Therefore, administration of a *CDK1* inhibitor may be an attractive strategy to treat patients with oxaliplatin‐resistant CRC.

## Graphical abstract

Oxaliplatin resistance is a thorny issue in the clinical treatment of colorectal cancer (CRC). The current study demonstrates that CDK1 facilitates oxaliplatin resistance in a cell cycle‐independent manner. CDK1 directly phosphorylates and ubiquitinates ACSL4 protein, resulting in ACSL4 degradation, thereby inhibiting ferroptosis and promoting oxaliplatin resistance. CDK1 blockade may be an attractive strategy to treat CRC patients with oxaliplatin resistance.

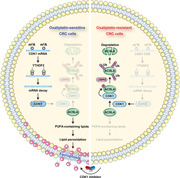

## 1 Introduction

Colorectal cancer (CRC) is the most common malignant tumor of the digestive tract that causes approximately 900 000 deaths annually and ranks third and second in global morbidity and mortality rates, respectively.^[^ ^1^ ^]^ Chemotherapy is the most indispensable and crucial strategy for CRC treatment.^[^ ^2^ ^]^ Oxaliplatin is a third‐generation platinum‐based antitumor drug; oxaliplatin‐based chemotherapy is mainly used as the first‐line treatment for advanced CRC and in adjuvant therapy after the complete resection of the primary tumor.^[^ ^3^ ^]^ Despite the initial efficacy of chemotherapy, approximately 50% of patients with stages II and III CRC develop resistance to oxaliplatin‐based adjuvant therapy,^[^ ^4^ ^]^ further increasing the difficulty in treatment. Therefore, elucidating the key molecules and potential mechanisms responsible for oxaliplatin resistance may aid in the development of new therapeutic strategies to overcome the current challenges in CRC treatment.

Ferroptosis is a new type of iron‐dependent programmed cell death, which is mechanistically and morphologically different from apoptosis, cell necrosis, and autophagy.^[^ ^5^ ^]^ It is triggered by the lethal accumulation of lipid peroxides on cellular membranes.^[^ ^6^ ^]^ Ferroptosis is dynamically controlled by oxidation and antioxidation systems involving several signaling pathways, such as the solute carrier family 7 member 11 (*SLC7A11*)/glutathione/glutathione peroxidase 4 (*GPX4*), *FSP1/CoQH2*, *DHODH/CoQH2*, and *GCH1/BH4* axes.^[^ ^7^ ^]^ Tumor cells can escape ferroptosis for uncontrolled disease progression and drug resistance, and pharmacological or genetic induction of ferroptosis can effectively restore the chemosensitivity of cells.^[^ ^8^, ^9^, ^10^ ^]^ MT‐1G contributes to sorafenib resistance in hepatocellular carcinoma, and its inhibition promotes glutathione depletion and ferroptosis, enhancing the anti‐tumor activity of sorafenib.^[^ ^11^ ^]^ Roblitinib, a fibroblast growth factor receptor 4 inhibitor, induces lipid peroxidation by regulating the β‐catenin*/TCF4/SLC7A11/FPN1* axis, thereby overcoming the intrinsic and acquired trastuzumab resistance in patients with *HER2*‐positive breast cancer.^[^ ^12^ ^]^

Lipid peroxidation, a free radical‐driven reaction that mainly affects polyunsaturated fatty acid‐containing phospholipids (PUFA‐PLs), is the prerequisite for ferroptosis induction due to the presence of bis‐allylic moieties in PUFAs.^[^ ^13^ ^]^ Acyl‐CoA synthetase long‐chain family 4 (ACSL4) is a key enzyme that mediates PUFA‐PL synthesis; specifically, it catalyzes the ligation of free PUFAs and converts them into PUFA‐CoA, followed by esterification and integration into phospholipids mediated by lysophosphatidylcholine acyltransferase 3 to form PUFA‐PL.^[^ ^14^ ^]^ Phosphorylation of ACSL4 T328 by protein kinase C (PKC)‐βII is critical for the activation of ACSL4, which leads to the formation of the lipid peroxidation–PKCβII–ACSL4 positive feedback loop that amplifies lipid peroxidation and induces ferroptosis.^[^ ^15^ ^]^ These data indicate that ACSL4 is a sensitive monitor and decisive contributor to ferroptosis. ACSL4 is regulated by several transcription factors and microRNAs (miRNAs), such as *YAP*,^[^ ^16^ ^]^ *miR‐23a‐3p*,^[^ ^17^ ^]^ and *miR‐424‐5p*;^[^ ^18^ ^]^ however, little is known about the posttranslational modification of ACSL4, especially ACSL4 protein turnover.

Cyclin‐dependent kinase 1 (CDK1), a highly conserved Ser/Thr protein kinase, plays a vital role in cell cycle progression.^[^ ^19^ ^]^ CDK1 also performs multifaceted functions independent of cell cycle regulation by affecting the chromatin structure, protein synthesis, cell morphology, and stemness.^[^ ^20^ ^]^ As a protein kinase, CDK1 directly binds to and phosphorylates its canonical and noncanonical substrates, thereby participating in various biological processes.^[^ ^21^ ^]^ Owing to its high throughput and efficiency, whole‐genome screening based on CRISPR/Cas9 gene editing technology is widely used to study disease mechanisms and drug development, providing a scientific basis for clinical treatment.^[^ ^22^ ^]^ Herein, we conducted in vitro and in vivo genome‐wide CRISPR/Cas9 loss‐of‐function screening to identify the key genes driving oxaliplatin resistance in CRC. In‐depth analysis revealed *CDK1* as an essential contributor to oxaliplatin resistance. ACSL4 was identified as a novel phosphorylation substrate of CDK1, and the blockade of *ACSL4*‐induced ferroptosis was indispensable for *CDK1* to drive drug resistance. Therefore, our data suggest *CDK1* as a potential target for oxaliplatin‐resistant CRC treatment.

## 2 Results

### 2.1 *CDK1* is Essential for Oxaliplatin Resistance in CRC

**Figure 1:**
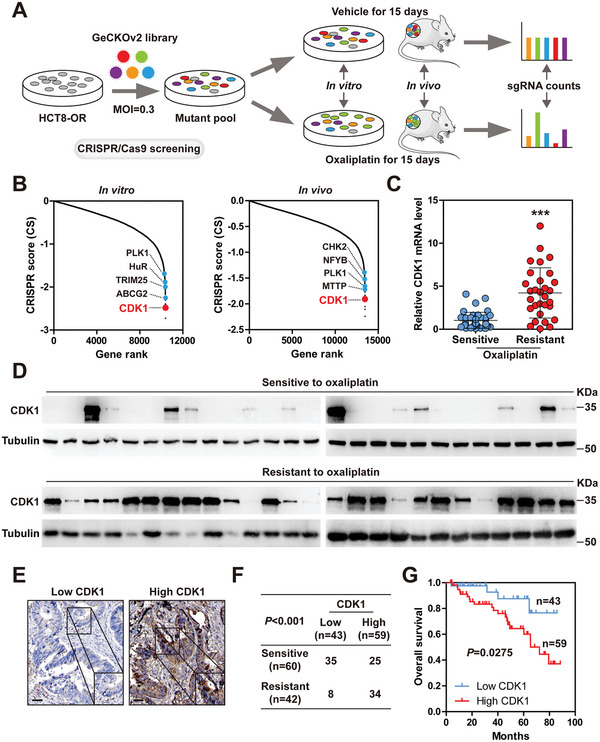
CDK1 is upregulated in colorectal cancer (CRC) with oxaliplatin resistance. A) The flow chart of CRISPR/Cas9 screening in vitro and in vivo. B) The CRISPR score (CS) of each gene in CRISPR/Cas9 screening. C) Quantitative reverse transcription‐polymerase chain reaction (qRT‐PCR) analysis of CDK1 mRNA levels in oxaliplatin‐sensitive (*n* = 39) and resistant (*n* = 30) CRC tissues. D) Immunoblotting (IB) assay analyzing the expression of CDK1 protein in oxaliplatin‐sensitive (*n* = 26) and resistant (*n* = 26) CRC tissues. E,F) Immunohistochemical (IHC) staining of CDK1 in paraffin‐embedded CRC tissues (*n* = 102), followed by analysis of the correlation between CDK1 and oxaliplatin resistance. Scale bars, 50 µm. G) The survival curve of CRC patients with low (*n* = 43) or high (*n* = 59) CDK1 levels. ^***^ *p* < 0.001, by two‐tailed unpaired Student's *t* test (C), chi‐square test (F), or log‐rank test (G).

To identify the key genes responsible for oxaliplatin resistance, we evaluated the intrinsic sensitivity of CRC cells to oxaliplatin. As shown in Figure S1A (Supporting Information), HCT8 and HT29 cells had lower IC50 values (0.453 × 10^−6^ m for HCT8 and 0.496 × 10^−6^ m for HT29 cells). HCT8 and HT29 cells were then exposed to cycles of gradually increasing concentrations of oxaliplatin for 9 months to establish oxaliplatin‐resistant cells (HCT8‐OR and HT29‐OR) (Figure S1C–F, Supporting Information). Subsequently, we conducted genome‐wide CRISPR/Cas9 loss‐of‐function screening using the GeCKO v2 human library containing 123 411 sgRNAs targeting 19 050 human genes, and both in vitro and in vivo screening were carried out to ensure the accuracy of the results (**Figure** **1**A). Theoretically, in the presence of oxaliplatin, sgRNAs targeting genes crucial to cell survival will be depleted. As expected, some genes reported to be associated with oxaliplatin resistance, such as PLK1, HuR, and CHK2, were also identified in our screening model (Figure 1B; Tables S1 and S2, Supporting Information). Given that CDK1 ranked high in both in vitro and in vivo screening and is more druggable, we chose it for subsequent investigation rather than other top candidates (Figure 1B; Tables S1 and S2, Supporting Information). The mRNA and protein levels of CDK1, but not other CDK family members, such as CDK2, CDK4, CDK6, and CDK9, were significantly upregulated in oxaliplatin‐resistant CRC tissues (Figure 1C,D; Figure S1G–K, Supporting Information). Next, we validated CDK1 expression in paraffin‐embedded tissues via immunohistochemical (IHC) staining. The results showed that 80.95% of patients with oxaliplatin resistance had high CDK1 expression (Figure 1E,F). Moreover, CRC patients with high CDK1 expression displayed a shorter overall time than those with low CDK1 expression (Figure 1G). These data suggest that the upregulation of CDK1 in CRC may be responsible for oxaliplatin resistance.

### 2.2 Knockout of *CDK1* Restores the Sensitivity of CRC Cells to Oxaliplatin

**Figure 2:**
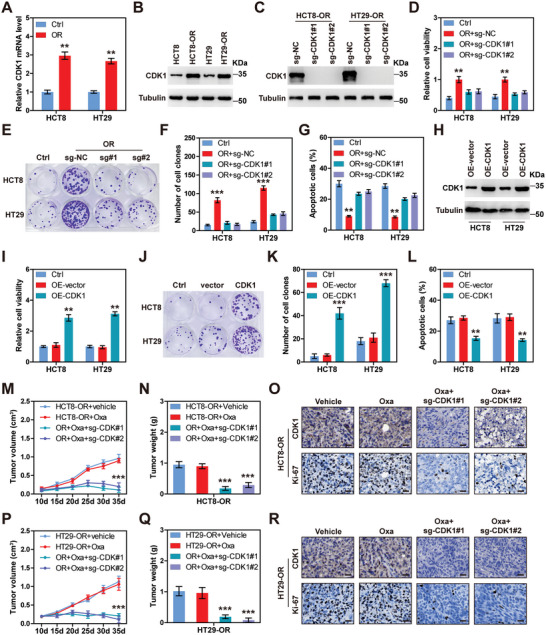
CDK1 knockout enhances the anti‐tumor effect of oxaliplatin. A) Quantitative reverse transcription‐polymerase chain reaction (qRT‐PCR) analysis of CDK1 mRNA levels in control and HCT8/HT29‐OR cells. B,C) Immunoblotting (IB) analysis of CDK1 protein expression in the indicated cell lines. D–G) CCK‐8, colony formation and flow cytometry testing cell viability, clonogenic capacity, and apoptosis in CDK1 knockout cell lines following 1 × 10^−6^ m oxaliplatin treatment for 48 h (CCK‐8 and apoptosis) and 14 days (colony formation). H) IB analysis of CDK1 protein expression in HCT8 and HT29 cells after 72 h of transfection with CDK1 plasmid. I–L) CCK‐8, colony formation, and flow cytometry testing cell viability, clonogenic capacity, and apoptosis in CDK1‐overexpressed HCT8 and HT29 cells following 1 × 10^−6^ m oxaliplatin treatment for 48 h (CCK‐8 and apoptosis) and 14 days (colony formation). M–R) Tumor volume and weight in the indicated groups (*n* = 5), followed by immunohistochemical (IHC) staining of CDK1 and Ki‐67 proteins. Scale bars, 50 µm. The data are shown as the mean ± SD (*n* = 3). ^**^ *p* < 0.01, ^***^ *p* < 0.001, by two‐tailed unpaired Student's *t* test (A), one‐way ANOVA (D,F,G,I,K,L,N,Q) or two‐way ANOVA (M,P) followed by Tukey's post hoc test. Ctrl, Control; OR, oxaliplatin resistance; NC, negative control; OE, overexpressed; Oxa, oxaliplatin.

Consistently, CDK1 mRNA and protein levels were higher in HCT8‐OR and HT29‐OR cells than in the control parental cells (**Figure** **2**A,B; Figure S2A,B, Supporting Information). Then, CDK1 was knocked out using CRISPR/Cas9 gene editing (Figure 2C). Cell counting kit (CCK)−8 assay revealed that CDK1 knockout significantly reduced the viability of HCT8‐OR and HT29‐OR cells following oxaliplatin treatment (Figure 2D). Likewise, the number of clones formed by resistant cells dropped almost to the level of parental cells after CDK1 knockout in the presence of oxaliplatin (Figure 2E,F). More apoptotic cells were observed in HCT8‐OR and HT29‐OR CDK1^−/−^ cells than in control cells after treatment with oxaliplatin (Figure 2G). In contrast, overexpression of CDK1 in HCT8 and HT29 cells conferred resistance to oxaliplatin, as illustrated by CCK8, colony formation, and apoptotic assays (Figure 2H–L). Furthermore, we subcutaneously injected HCT8‐OR and HT29‐OR cells into NOD/SCID mice, followed by an intraperitoneal injection of oxaliplatin. The results showed that HCT8‐OR and HT29‐OR‐bearing mice did not respond to oxaliplatin; however, when CDK1 was knocked out, the tumor mass was significantly reduced following oxaliplatin treatment, which was further verified by IHC staining of the Ki‐67 (a proliferation marker) (Figure 2M–R). In addition, the oxaliplatin‐sensitive HCT8 cells were also subcutaneously injected into mice. As expected, oxaliplatin markedly retarded the growth of HCT8‐derived tumors, while this effect was blocked by CDK1 overexpression (Figure S2C–E, Supporting Information).

Taken together, these results suggest that CDK1 depletion overcomes oxaliplatin resistance in CRC.

### 2.3 *CDK1* Expression is Upregulated by N6‐methyladenosine (m^6^A) Hypomethylation

To understand how CDK1 is upregulated, HCT8‐OR and HT29‐OR cells were treated with 5‐Azacytidine (5‐Aza‐dC, a DNA methyltransferase inhibitor) and Trichostatin A (TSA, a histone deacetylase inhibitor). Quantitative reverse transcription‐polymerase chain reaction (qRT‐PCR) revealed that CDK1 mRNA was unaltered (Figure S3A,B, Supporting Information), suggesting that DNA methylation and histone acetylation are not involved in the regulation of CDK1. And silencing of some transcription factors, such as TP53 and HIF‐1α, had slight effects on CDK1 expression (Figure S3C,D, Supporting Information). In addition, no copy number variation was observed between oxaliplatin‐resistant and oxaliplatin‐sensitive CRC tissues (Figure S3E, Supporting Information).

**Figure 3:**
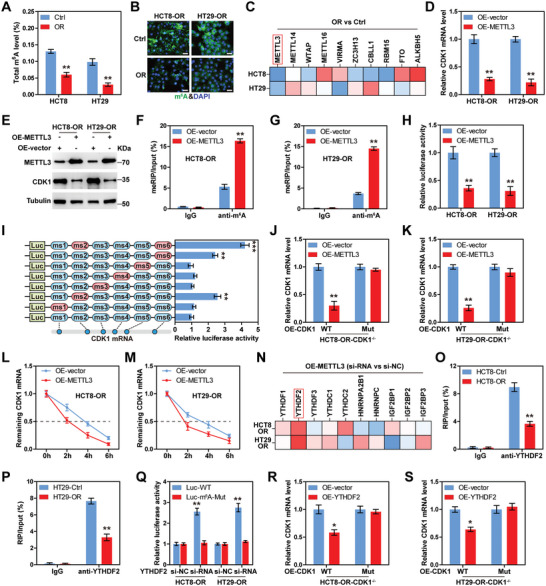
CDK1 is regulated by m^6^A modification. A,B) Total m^6^A levels in HCT8‐OR and HT29‐OR cells tested by colorimetric and IF assays. Scale bars, 50 µm. C) Quantitative reverse transcription‐polymerase chain reaction (qRT‐PCR) analysis of the indicated gene expression in HCT8‐OR and HT29‐OR cells. D,E) qRT‐PCR analysis of the effect of METTL3 overexpression on CDK1 mRNA expression. F,G) meRIP assay analyzing the enrichment of m^6^A on CDK1 mRNA after METTL3 overexpression. H,I) The activity of m^6^A on CDK1 mRNA was analyzed by luciferase reporter assay with wild‐type and mutated vectors. J,K) qRT‐PCR analysis of CDK1 mRNA levels in CDK1 knockout cells overexpressing METTL3 and wild‐type or mutated CDK1. L,M) qRT‐PCR analysis of CDK1 mRNA levels in METTL3‐overexpressed HCT8‐OR and HT29‐OR cells treated with 5 µg mL^−1^ actinomycin D for the indicated time. N) qRT‐PCR analysis of CDK1 mRNA levels in METTL3‐overexpressed HCT8‐OR and HT29‐OR cells transfected with the indicated siRNAs. O,P) RIP assays detecting the enrichment of YTHDF2 on CDK1 mRNA. Q) Luciferase reporter assay testing the effect of YTHDF2 silencing on m^6^A activity of CDK1 mRNA. R,S) qRT‐PCR analysis of CDK1 mRNA levels in CDK1 knockout cells overexpressing YTHDF2 and wild‐type or mutated CDK1. The data are shown as the mean ± SD (*n* = 3). ^*^ *p* < 0.05, ^**^ *p* < 0.01, ^***^ *p* < 0.001, by two‐tailed unpaired Student's *t* test (A,D,H), one‐way ANOVA (I) or two‐way ANOVA (F,G,J–M,O–S) followed by Tukey's post hoc test. Ctrl, Control; OR, oxaliplatin resistance; NC, negative control; OE, overexpressed; ms, m^6^A site; WT, wild‐type; Mut, mutation.

Recent evidence has shown that m^6^A plays a prominent role in RNA metabolism;^[^ ^23^ ^]^ therefore, we wondered whether CDK1 was regulated by m^6^A. As shown in **Figure** **3**A,B, the total m^6^A levels in HCT8‐OR and HT29‐OR cells were significantly reduced compared to those in the parental cells. The levels of some known m^6^A writers and erasers were tested, and the results showed that only m^6^A “writer” METTL3 was markedly downregulated in both HCT8‐OR and HT29‐OR cells (Figure 3C). Reinforced expression of METTL3 reduced CDK1 mRNA and protein levels (Figure 3D,E; Figure S3F,G, Supporting Information), along with increased m^6^A enrichment in CDK1 mRNA (Figure 3F,G; Figure S3H, Supporting Information). We then constructed a luciferase reporter by inserting the full‐length of CDK1 mRNA behind the luciferase gene of the pmirGLO vector (Figure S3I, Supporting Information), followed by transfection into cells, the results showed that the luciferase activity of CDK1 mRNA was dramatically decreased following METTL3 overexpression (Figure 3H). Using SRAMP,^[^ ^24^ ^]^ a sequence‐based m^6^A modification site predictor, we found six putative m^6^A motifs in CDK1 mRNA (Figure S3J, Supporting Information); step‐by‐step mutation was carried out by replacing adenosine with thymine (Figure S3J, Supporting Information). The results of the luciferase reporter assay showed that mutation of 499–503 (ms2) and 1669–1673 sites (ms6) on CDK1 mRNA increased luciferase activity (Figure 3I), suggesting that these two m^6^A motifs are required for METTL3‐mediated CDK1 regulation. Consistently, METTL3 notably reduced CDK1 expression in HCT8‐OR and HT29‐OR CDK1^−/−^ cells reintroducing wild‐type CDK1, but not CDK1 with m^6^A mutation of ms2 and ms6 (Figure 3J,K).

Moreover, the half‐life of CDK1 mRNA was significantly shortened following METTL3 overexpression (Figure 3L,M). We then evaluated which m^6^A readers participated in this process. As shown in Figure 3N, only silencing of YTHDF2 rescued the reduced CDK1 mRNA level caused by METTL3 overexpression. Consistently, less YTHDF2 enrichment on CDK1 mRNA was observed in oxaliplatin‐resistant cells than in parental cells (Figure 3O,P). However, there was no difference in YTHDF2 expression between parental and oxaliplatin‐resistant CRC cells (Figure S3K–M, Supporting Information). Knockdown of YTHDF2 increased the luciferase activity of the wild‐type reporter, but not the m^6^A‐mutated reporter (mutation of ms2 and ms6) (Figure 3Q). Likewise, YTHDF2 overexpression decreased CDK1 expression in HCT8‐OR and HT29‐OR CDK1^−/−^ cells reintroducing wild‐type CDK1, but not m^6^A‐mutated CDK1 (mutation of ms2 and ms6) (Figure 3R,S). Taken together, these findings suggest that CDK1 is modified by METTL3‐mediated m^6^A, followed by mRNA decay via YTHDF2‐dependent recognition.

### 2.4 CDK1 Binds to and Degrades ACSL4

**Figure 4:**
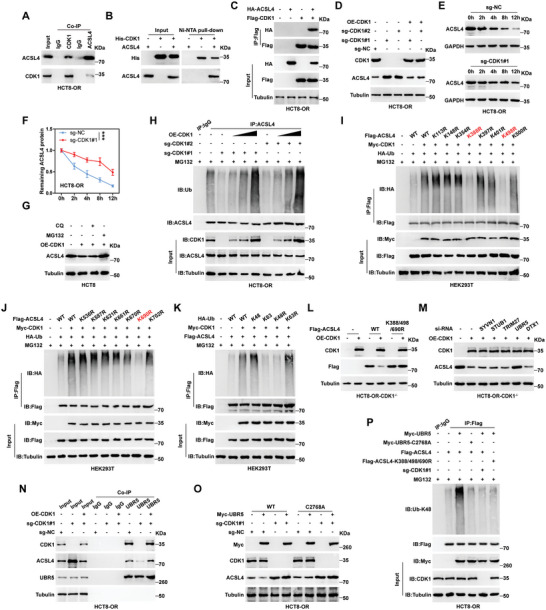
CDK1 reduces ACSL4 protein stability. A) Co‐immunoprecipitation (Co‐IP) assay testing the endogenous interaction between CDK1 and ACSL4. B) His pull‐down assay testing the direct binding of CDK1 to ACSL4 in vitro. C) HCT8‐OR cells transfected with the labeled plasmids for 48 h, followed by IP coupled with immunoblotting (IB) assays using the indicated antibodies. D) IB assay testing the expression of CDK1 and ACSL4 proteins in CDK1 knockout HCT8‐OR cells. E,F) IB assay testing ACSL4 expression in CDK1 knockout HCT8‐OR cells treated with 100 µg mL^−1^ cycloheximide for the indicated time. G) IB assay testing ACSL4 expression in CDK1‐overexpressing HCT8 cells treated with 5 × 10^−6^ m MG132 or 10 × 10^−6^ m chloroquine for 12 h. H) IP assay using anti‐ACSL4 antibody, followed by IB assay with the indicated antibodies in HCT8‐OR cells treated with 20 × 10^−6^ m MG132 for 6 h. I–K) HEK293T cells were transfected with the indicated vectors for 48 h, followed by IP coupled with IB assays using the indicated antibodies. L,M) IB assays detecting the indicated protein levels in CDK1 knockout HCT8‐OR cells transfected with the indicated vectors or siRNAs for 48 h. N) IP assay using anti‐UBR5 antibody, followed by IB assay with the indicated antibodies in HCT8‐OR cells. O) IB assay detecting the indicated protein levels in CDK1 knockout HCT8‐OR cells transfected with wild‐type or mutant UBR5 plasmid for 48 h. P) HCT8‐OR cells were transfected with the indicated vectors for 48 h, followed by IP coupled with IB assays using the indicated antibodies. The data are shown as the mean ± SD (*n* = 3). ^***^ *p* < 0.001, by two‐way ANOVA followed by Tukey's post hoc test (F). NC, negative control; OE, overexpressed; WT, wild‐type; CQ, chloroquine.

Given that CDK1 is a typical driver of G2/M phase transition, we then tested the cell cycle progression in oxaliplatin‐resistant and ‐sensitive cells. Unexpectedly, compared with control cells, the cell cycle of HCT8‐OR and HT29‐OR cells was not accelerated; instead, a slight G2/M phase arrest was observed in HT29‐OR cells (Figure S3N, Supporting Information), suggesting that CDK1 drives oxaliplatin resistance in a cell cycle‐independent manner. To clarify how CDK1 functions in oxaliplatin resistance, we enriched CDK1 binding proteins, followed by mass spectrometry (Figure S4A, Supporting Information). Some known CDK1 binding partners were identified, such as CCNB1 and HNRNPUL1 (Figure S4A; Table S3, Supporting Information). Of note, ACSL4, a key factor in the execution of ferroptosis related to drug resistance, was found to be immunoprecipitated by CDK1 (Figure S4A; Table S3, Supporting Information). We then inferred that CDK1 contributed to oxaliplatin resistance via ACSL4. Endogenous co‐immunoprecipitation (Co‐IP) revealed that CDK1 and ACSL4 bound to each other in both HCT8‐OR and HT29‐OR cells (**Figure** **4**A; Figure S4B, Supporting Information). And the co‐localization of CDK1 and ACSL4 was observed in immunofluorescence staining (Figure S4C, Supporting Information). The Ni‐NTA pull‐down assay showed that CDK1 directly interacted with ACSL4 in vitro (Figure 4B). Moreover, we transfected labeled exogenous CDK1 and ACSL4 proteins into CRC cells and found binding between them (Figure 4C; Figure S4D, Supporting Information). Intriguingly, the expression of ACSL4 protein, but not mRNA, was notably increased in HCT8‐OR and HT29‐OR CDK1^−/−^ cells compared to control cells; however, reintroduction of CDK1 abolished the above effects (Figure 4D; Figure S4E–G, Supporting Information). The half‐life of the ACSL4 protein was significantly prolonged after CDK1 knockout (Figure 4E,F; Figure S4H, Supporting Information), and the decrease in ACSL4 caused by CDK1 overexpression was rescued by MG132 (a proteasome inhibitor) rather than by chloroquine (an autophagy inhibitor) (Figure 4G; Figure S4I–K, Supporting Information), indicating that the ubiquitin‐proteasome system is responsible for CDK1‐mediated degradation of ACSL4.

As expected, the endogenous ubiquitination level of ACSL4 was significantly decreased by CDK1 knockout but was increased by CDK1 reintroduction in a dose‐dependent manner (Figure 4H; Figure S4L, Supporting Information). By analyzing the UbiBrowser database,^[^ ^25^ ^]^ we found 15 known lysine ubiquitination sites on the ACSL4 protein. A step‐by‐step mutation was conducted by replacing lysine with arginine, followed by ubiquitination analysis. The results showed that only K388R, K498R, and K690R could block the increased ubiquitination level of ACSL4 caused by CDK1 (Figure 4I,J). Moreover, the branched ubiquitin chains of K48, but not K63, mediated the degradation of ACSL4 by CDK1 (Figure 4K). Overexpression of CDK1 in CDK1^−/−^ cells reduced ACSL4 protein levels, but this effect disappeared after mutation of K388, K498, and K690 on ASCL4 (Figure 4L; Figure S4M,N, Supporting Information).

Given that CDK1 is not an E3 ubiquitin ligase, we wondered which ubiquitin ligases were involved in the regulation of ACSL4 by CDK1. The intersection results of the CDK1‐enriched mass spectrum and UbiBrowser‐predicted E3 ligases for ACSL4 showed that five E3 ligases might concurrently bind to CDK1 and ACSL4 (Figure S4O, Supporting Information). We then silenced them one by one and found that only knockdown of UBR5 was able to counteract the reduced ACSL4 levels caused by CDK1 reintroduction in CDK1^−/−^ cells (Figure 4M; Figure S4P–R, Supporting Information). The endogenous Co‐IP results showed that CDK1 and ACSL4 were both immunoprecipitated by UBR5, and knockout of CDK1 decreased, while reintroduction of CDK1 increased the binding of UBR5 to ACSL4 in both HCT8‐OR and HT29‐OR cells (Figure 4N; Figure S4S, Supporting Information). Moreover, overexpression of wild‐type UBR5, but not the ubiquitin ligase‐deficient mutant (C2768A), significantly reduced ACSL4 protein expression; however, this effect was abolished in the absence of CDK1 (Figure 4O; Figure S4T–V, Supporting Information). Consistently, the K48‐linked ubiquitination levels of ACSL4 were increased by overexpression of UBR5 rather than UBR5‐C2768A, and either knockout of CDK1 or mutation of K388/K498/K690 in ACSL4 abrogated the above effects (Figure 4P; Figure S4W, Supporting Information). In addition, the in vitro ubiquitination assay showed that UBR5, but not UBR5‐C2768A, could directly ubiquitinate ACSL4 (Figure S4X, Supporting Information).

Taken together, these results indicate that CDK1 physically binds to ACSL4 and increases the K48‐linked polyubiquitination of ACSL4 in a UBR5‐dependent manner, leading to ACSL4 protein degradation in oxaliplatin‐resistant CRC cells.

### 2.5 CDK1 Directly Phosphorylates ACSL4 at S447

**Figure 5:**
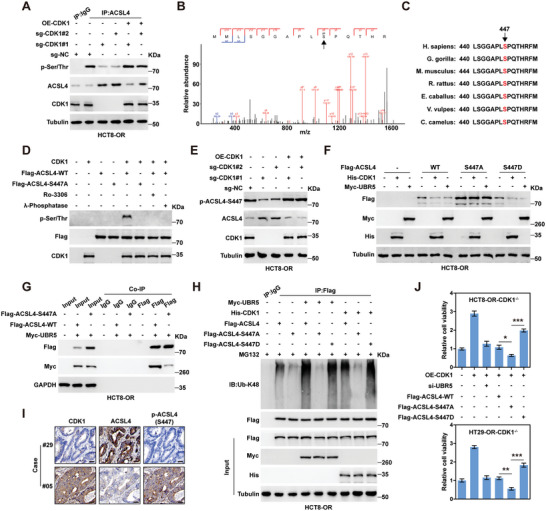
ACSL4 is phosphorylated by CDK1 at S447. A) Immunoprecipitation (IP) coupled with immunoblotting (IB) assays using the indicated antibodies testing the effect of CDK1 on ACSL4 phosphorylation. B) Mass spectrometry identifying the phosphorylation of ACSL4 S447. C) Evaluation of the conservation of ACSL4 S447 among different species. D) In vitro phosphorylation assay using the purified proteins, followed by IB assay using the indicated antibodies. E,F) IB assay using the indicated antibodies in HCT8‐OR cells transfected with the indicated vectors for 48 h. G,H) IP coupled with IB assays using the indicated antibodies in HCT8‐OR cells transfected with the indicated vectors for 48 h. I) Immunohistochemical (IHC) staining of CDK1, ACSL4, and p‐ACSL4‐S447 in paraffin‐embedded colorectal cancer (CRC) tissues. Scale bars, 50 µm. J) CCK‐8 assay testing cell viability in CDK1 knockout cells transfected with the indicated vectors after 1 × 10^−6^ m oxaliplatin treatment for 72 h. The data are shown as the mean ± SD (*n* = 3). ^*^ *p* < 0.05, ^**^ *p* < 0.01, ^***^ *p* < 0.001, by one‐way ANOVA followed by Tukey's post hoc test (J). NC, negative control; OE, overexpressed; WT, wild‐type.

Considering that CDK1 is a Ser/Thr protein kinase, we wondered whether ACSL4 was phosphorylated by CDK1. As shown in **Figure** **5**A and Figure S5A (Supporting Information), the Ser/Thr phosphorylation levels of ACSL4 were dramatically decreased in HCT8‐OR and HT29‐OR CDK1^−/−^ cells compared to control cells, accompanied by an increase in total ACSL4 protein, but the above effects were abolished after CDK1 reintroduction. Using the Scansite online tool,^[^ ^26^ ^]^ we found that CDK1 might phosphorylate ACSL4 at S57 and S447. The exogenous IP assay was conducted in CDK1‐overexpressing cells, followed by mass spectrometric analysis of phosphorylation sites, and the results showed that S447, but not S57, was identified (Figure 5B). ACSL4 S447 is highly conserved across species, as illustrated by sequence alignment analysis (Figure 5C). The results of in vitro phosphorylation assay showed that CDK1 phosphorylated ACSL4, but this effect disappeared after mutation of serine to alanine at position 477 (a phospho‐deficient mutant) or treatment with the CDK1 inhibitor RO‐3306 (Figure 5D). We then generated an antibody specifically targeting p‐ACSL4 S447 and verified its effectiveness (Figure S5B, Supporting Information). As expected, the levels of p‐ACSL4 S447 decreased after CDK1 knockout, and re‐expression of CDK1 restored p‐ACSL4 S447 levels (Figure 5E; Figure S5C,D, Supporting Information). Overexpression of CDK1 or UBR5 reduced ACSL4 levels in cells overexpressing ACSL4‐WT or ACSL4‐S447D (a phospho‐mimetic mutant), but not in cells overexpressing the phospho‐deficient mutant ACSL4‐S447A (Figure 5F; Figure S5E–G, Supporting Information). Moreover, ACSL4‐S447A blocked the interaction between UBR5 and ACSL4 in both HCT8‐OR and HT29‐OR cells (Figure 5G; Figure S5H, Supporting Information). Consistently, the K48‐linked ubiquitination levels of ACSL4 were increased by UBR5 or CDK1 in cells overexpressing ACSL4‐WT or ACSL4‐S447D; however, the above effect was not observed in cells overexpressing ACSL4‐S447A (Figure 5H; Figure S5I, Supporting Information). Importantly, CDK1 expression was negatively correlated with ACSL4 levels but positively correlated with p‐ACSL4 S447 levels in clinical CRC tissues (Figure 5I; Figure S5J,K, Supporting Information). Functionally, overexpression of ACSL4‐S447A significantly reduced cell viability compared to overexpression of ACSL4‐WT or ACSL4‐S447D in HCT8‐OR and HT29‐OR CDK1^−/−^ cells re‐expressing CDK1 following oxaliplatin treatment (Figure 5J). In summary, the above data suggest that ACSL4 S447 phosphorylation is required for CDK1 degradation of ACSL4 via UBR5 in oxaliplatin‐resistant CRC cells.

### 2.6 *CDK1* Confers Oxaliplatin Resistance by Blocking ACSL4‐mediated Ferroptosis

**Figure 6:**
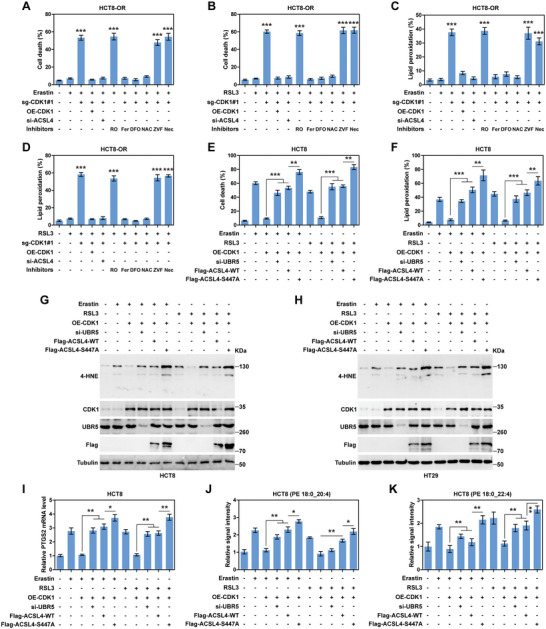
CDK1 represses ACSL4‐mediated ferroptosis. A–D) The indicated vectors or siRNAs were transfected into HCT8‐OR cells treated with 1 × 10^−6^ m erastin, 100 × 10^−9^ m RSL3, 2 × 10^−6^ m RO‐3306, 5 × 10^−6^ m ferrostatin‐1, 5 × 10^−6^ m deferoxamine, 5 × 10^−3^ m *N*‐acetyl‐cysteine, 10 × 10^−6^ m Z‐VAD‐FMK or 2 × 10^−6^ m necrostatin‐1 for 20 h, followed by detection of cell death and lipid peroxidation. E,F) The indicated vectors or siRNAs were transfected into HCT8 cells treated with 2 × 10^−6^ m erastin, 200 × 10^−9^ m RSL3 for 20 h, followed by detection of cell death and lipid peroxidation. G,H) Immunoblotting (IB) assay analyzing the indicated protein levels in HCT8 and HT29 cells transfected with the indicated vectors or siRNAs and treated with 2 × 10^−6^ m erastin or 200 × 10^−9^ m RSL3 for 20 h. I) Quantitative reverse transcription‐polymerase chain reaction (qRT‐PCR) analysis of PTGS2 mRNA levels in HCT8 cells transfected with the indicated vectors or siRNAs and treated with 2 × 10^−6^ m erastin or 200 × 10^−9^ m RSL3 for 20 h. J,K) Mass spectrometric analysis of the signal intensities of PE 18:0_20:4 and PE 18:0_22:4 in HCT8 cells transfected with the indicated vectors or siRNAs and treated with 2 × 10^−6^ m erastin or 200 × 10^−9^ m RSL3 for 20 h. The data are shown as the mean ± SD (*n* = 3). ^*^ *p* < 0.05, ^**^ *p* < 0.01, ^***^ *p* < 0.001, by one‐way ANOVA followed by Tukey's post hoc test (A–F, I–K). OE, overexpressed; WT, wild‐type. RO, RO‐3306; Fer, ferrostatin‐1; DFO, deferoxamine; NAC, *N*‐acetyl‐cysteine; ZVF, Z‐VAD‐FMK; Nec, necrostatin‐1.

As shown in Figure S6A–D (Supporting Information), HCT8‐OR and HT29‐OR cells were more resistant to the ferroptosis inducer erastin or RSL3 than their parental cells. Noticeably, CDK1 knockout or RO‐3306 treatment significantly increased the susceptibility of HCT8‐OR and HT29‐OR cells to erastin or RSL3, accompanied by high levels of lipid peroxidation; however, re‐expression of CDK1, silencing of ACSL4 or treatment with rosiglitazone (an ACSL4 inhibitor) abolished these effects (**Figure** **6**A–D; Figure S6E–J, Supporting Information). The increased lipid peroxidation and cell death caused by CDK1 knockout in erastin‐ or RSL3‐treated cells were effectively blocked by the addition of ferrostatin‐1 (a ferroptosis inhibitor), deferoxamine (an iron chelator), or *N*‐acetyl‐cysteine (a ROS scavenger), but not by the addition of Z‐VAD‐FMK (a pan‐caspase inhibitor) or necrostatin‐1 (a necroptosis inhibitor) (Figure 6A–D; Figure S6E–H, Supporting Information). In contrast, CDK1 overexpression decreased cell death and lipid peroxidation caused by erastin or RSL3 in HCT8 and HT29 cells, which were significantly rescued by UBR5 knockdown or ACSL4‐WT overexpression, especially after ACSL4‐S447A overexpression (Figure 6E,F; Figure S6K,L, Supporting Information). The above results were further verified by the detection of the ferroptosis markers 4‐hydroxy‐2‐nonenal (4‐HNE) and PTGS2 (Figure 6G–I; Figure S6M–O, Supporting Information). Consistently, the increased levels of PUFA‐PLs, such as phosphatidylethanolamine (PE) 18:0_20:4 and PE 18:0_22:4, induced by erastin or RSL3 were blocked by CDK1 overexpression; however, these effects were also abolished by UBR5 silencing or ACSL4‐WT overexpression, especially by ACSL4‐S447A overexpression (Figure 6J,K; Figure S6P,Q, Supporting Information). Altogether, these data demonstrate that the inhibition of ACSL4‐driven lipid peroxidation and ferroptosis is essential for CDK1 to promote oxaliplatin resistance in CRC.

### 2.7 CDK1 Inhibitor Overcomes Oxaliplatin Resistance in Cell/Patient‐Derived Xenograft (CDX/PDX) Models

**Figure 7:**
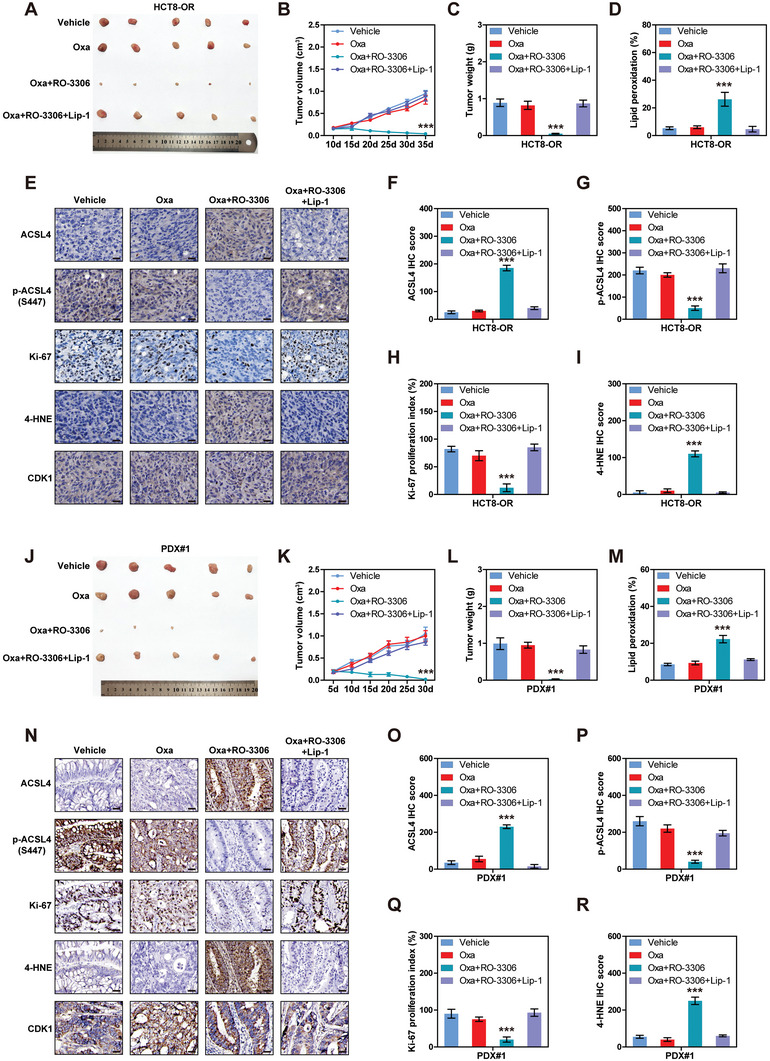
CDK1 inhibitor restores the sensitivity of oxaliplatin in vivo. A–C) The image, volume, and weight of tumor generated by HCT8‐OR injection in the indicated groups (*n* = 5). D) Flow cytometry detecting lipid peroxidation levels of HCT8‐OR tumor cells isolated from mice in the indicated groups. E–I) Immunohistochemical (IHC) staining of CDK1, ACSL4, p‐ACSL4‐S447, Ki‐67 and 4‐HNE in tumor tissues generated by HCT8‐OR injection in the indicated groups. Scale bars, 50 µm. J–L) The image, volume, and weight of tumor generated by xenotransplantation of clinical colorectal cancer (CRC) tissues (PDX#1) in the indicated four groups. M) Flow cytometry detecting lipid peroxidation levels of tumor cells isolated from mice in PDX#1 models (*n* = 5). N–R) IHC staining of CDK1, ACSL4, p‐ACSL4‐S447, Ki‐67 and 4‐HNE in tumor tissues generated by xenotransplantation of clinical CRC tissues (PDX#1) in the indicated groups. Scale bars, 50 µm. ^***^ *p* < 0.001, by one‐way ANOVA (C,D,F–I,L,M,O–R) or two‐way ANOVA (B,K) followed by Tukey's post hoc test. Oxa, oxaliplatin; Lip‐1, liproxstatin‐1.

Lastly, preclinical evaluation of the effects of CDK1 inhibitor on oxaliplatin resistance was carried out using animal models. For the CDX models, NOD/SCID mice were subcutaneously injected with HCT8‐OR or HT29‐OR cells, followed by administration of oxaliplatin, RO‐3306, and liproxstatin‐1, as shown in Figure S7A (Supporting Information). All mice survived at the end of the treatment, and no mouse exhibited severe loss of body weight (>15%) or evidence of infections or wounds (Figure S7B,C, Supporting Information), suggesting that the combination of the above drugs has no apparent toxicity in vivo. HCT8‐OR/HT29‐OR‐derived tumors were insensitive to oxaliplatin, and when combined with RO‐3306, tumor mass was substantially reduced, and individual tumors were almost undetectable; however, these anti‐tumor effects were completely abolished by the addition of liproxstatin‐1, a ferroptosis inhibitor (**Figure** **7**A–C; Figure S7D–F, Supporting Information). Consistently, higher lipid peroxidation, ACSL4, 4‐HNE, PTGS2, and Fe^2+^, and lower p‐ACSL4 S447 and Ki‐67 were observed in the oxaliplatin+RO‐3306 group than in the other three groups (Figure 7D–I; Figure S7G–R, Supporting Information).

Further, we established PDX models with better clinical relevance by using two clinically oxaliplatin‐resistant CRC tissues (Figure S8A; Table S4, Supporting Information), followed by treatment with oxaliplatin, RO‐3306, and liproxstatin‐1. Similarly, no mouse exhibited a severe loss of body weight (>15%) or evidence of infection or wounds (Figure S8B,C, Supporting Information). There was no statistical difference in tumor size between the control and oxaliplatin groups, whereas the tumor was dramatically downsized in the oxaliplatin+RO‐3306 group, and the addition of liproxstatin‐1 significantly restored tumor growth (Figure 7J–L; Figure S8D–F, Supporting Information). Compared with the control or oxaliplatin alone, oxaliplatin combined with RO‐3306 increased lipid peroxidation, ACSL4, 4‐HNE, PTGS2, and Fe^2+^ levels, along with a decrease in p‐ACSL4 S447 and Ki‐67 expression; however, these effects were counteracted after liproxstatin‐1 addition (Figure 7M–R; Figure S8G–R, Supporting Information).

Collectively, these results suggest that CDK1 inhibitors synergistically enhance the antitumor effect of oxaliplatin in CRC with oxaliplatin resistance.

## 3 Discussion

**Figure 8:**
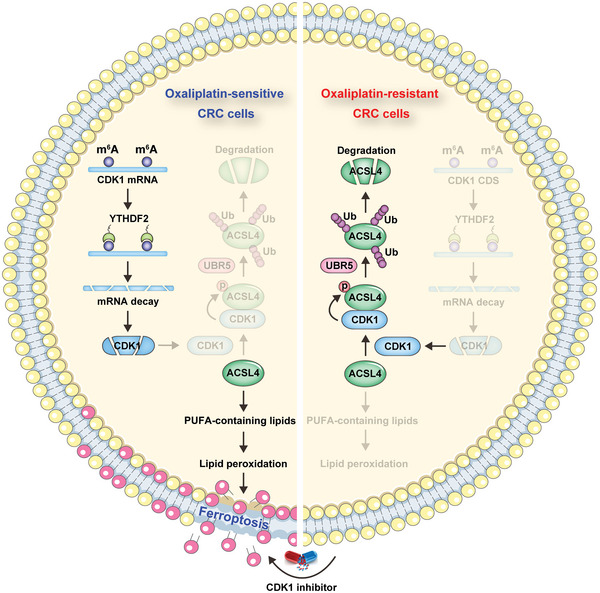
Summary diagram for the role of CDK1 in oxaliplatin resistance in colorectal cancer (CRC). m^6^A modification on CDK1 mRNA is decreased in oxaliplatin‐resistant CRC cells, resulting in increasing CDK1 mRNA stability. Then, CDK1 binds to and phosphorylates ACSL4, followed by UBR5‐mediated ACSL4 polyubiquitination and degradation, leading to blocking the biosynthesis of polyunsaturated fatty acid containing lipids, ultimately inhibiting lipid peroxidation and ferroptosis. Administration in combination with CDK1 inhibitor can effectively restore the sensitivity of CRC patients to oxaliplatin.

Oxaliplatin resistance is a thorny issue in the clinical treatment of CRC. In the present study, using genome‐wide CRISPR/Cas9 loss‐of‐function screening both in vitro and in vivo, we identified CDK1 as a key player in oxaliplatin resistance. CDK1 is significantly overexpressed in oxaliplatin‐resistant CRC and is closely linked to poor prognosis. Further investigations revealed that CDK1 contributes to oxaliplatin resistance by blocking ferroptosis via inhibition of ACSL4. CDK1 directly interacted with and degraded ACSL4 by recruiting E3 ubiquitin ligase UBR5, and this process depended on the phosphorylation of ACSL4 at S447 (**Figure** **8**). The in vitro and in vivo models showed that genetic and pharmacological blockade of CDK1 restored the anti‐tumor effect of oxaliplatin. Therefore, our findings uncover an intimate relationship between CDK1 and oxaliplatin resistance, which provides a preclinical rationale for targeting CDK1 as a therapeutic strategy for patients with oxaliplatin‐resistant CRC.

CDK1, the founding member of the cyclin‐dependent protein kinase (CDK) family, is frequently overexpressed in various human cancers and is strongly associated with malignant phenotypes and poor prognosis.^[^ ^27^ ^]^ Evidence suggests that the upregulation of CDK1 is due to copy number variation.^[^ ^28^, ^29^ ^]^ However, no statistical difference in the copy number of CDK1 was observed between oxaliplatin‐resistant and oxaliplatin‐sensitive CRC tissues, suggesting the existence of other mechanisms that regulate CDK1. m^6^A, the most common modification of eukaryotic RNA, is reversibly and dynamically controlled by m^6^A methylases (writers) and demethylases (erasers).^[^ ^30^ ^]^ The m^6^A writer METTL3, the sole catalytic subunit of the m^6^A modification system, is widely recognized to be critical for cancer development and progression,^[^ ^31^ ^]^ and it has been reported as an inhibitor or contributor of drug resistance depending on the context.^[^ ^32^ ^]^ Herein, we found that total m^6^A levels were reduced in oxaliplatin‐resistant CRC, which was attributed to METTL3 downregulation, suggesting that METTL3 may inhibit oxaliplatin resistance in CRC. Furthermore, we found that CDK1 was a novel target of METTL3, and CDK1 mRNA was modified by m^6^A via METTL3. The fate of m^6^A‐modified RNA is determined by m^6^A‐binding proteins (readers), the most prominent members of which are the YT521‐B homology (YTH) family, including YTHDF1/2/3 and YTHDC1/2.^[^ ^33^ ^]^ YTHDF1, YTHDF3, and YTHDC2 are closely related to RNA translation efficiency. YTHDC1 controls m^6^A‐mediated selective splicing, and YTHDF2 has a profound role in the regulation of RNA stability.^[^ ^34^ ^]^ In this study, overexpression of METTL3 reduced CDK1 mRNA stability, whereas knockdown of YTHDF2, but not other m^6^A readers, blocked this effect, suggesting that YTHDF2 recognizes the m^6^A‐modified CDK1 mRNA installed by METTL3, followed by CDK1 mRNA decay, resulting in CDK1 downregulation. Therefore, our data unveil a close link between m^6^A and oxaliplatin resistance in CRC. Further investigations are needed to elucidate the specific causes of METTL3 downregulation in the context of oxaliplatin resistance.

Another important finding was that ACSL4 is a novel phosphorylation substrate of CDK1. CDK1 binds to and degrades ACSL4 via direct phosphorylation. Phosphorylation is an ideal switch for regulating protein–protein interactions that control ubiquitin attachment and removal; phosphorylation‐specific protein ubiquitination, often known as phosphodegron recognition, is the key mechanism of tumorigenesis and progression.^[^ ^35^ ^]^ For instance, PROX1, a key factor for tumor metabolic plasticity, is phosphorylated by AMPK at S79, resulting in the recruitment of CUL4‐DDB1 ubiquitin ligase to promote PROX1 degradation.^[^ ^36^ ^]^ SCF^FBXO22^ was reported to be responsible for BAG3 ubiquitination and degradation, which requires ERK‐mediated phosphorylation of BAG3 at S377.^[^ ^37^ ^]^ Likewise, some proteins related to tumor progression were also shown to be phosphorylated and ubiquitinated by CDK1, such as p27,^[^ ^38^ ^]^ EZH2,^[^ ^39^ ^]^ and kindlin.^[^ ^40^ ^]^ Here, we found that ACSL4 is phosphorylated by CDK1 at S447, followed by UBR5‐mediated K48‐linked polyubiquitination at K388/K498/K690, suggesting that ACSL4 degradation requires a phosphodegron created by CDK1 for UBR5 recognition. Moreover, overexpression of ACSL4, especially the phospho‐deficient mutant ACSL4‐S447A, markedly abrogated the enhanced resistance to oxaliplatin caused by CDK1 overexpression, and the in vivo effect of CDK1 inhibition on restoring oxaliplatin sensitivity was antagonized by a ferroptosis inhibitor, indicating that CDK1 confers oxaliplatin resistance by repressing ACSL4‐mediated ferroptosis. Given that ACSL4 is the key execution factor of ferroptosis, further studies are warranted to clarify the factors that elevate ACSL4 at the transcriptional or posttranscriptional level, possibly involving some transcriptional activators or deubiquitinases, which may uncover natural suppressors of tumor development and drug resistance.

Strikingly, preclinical CDX and PDX models showed that RO‐3306, a selective CDK1 inhibitor, had a remarkable effect of restoring the sensitivity of CRC cells to oxaliplatin. Recent studies have demonstrated that the CDK1 inhibitor has targeted selectivity, which is reflected in its killing effect on tumor cells being four times that of normal cells.^[^ ^41^ ^]^ No significant side effects were observed after CDK1 inhibitor administration in vivo.^[^ ^42^, ^43^ ^]^ Consistent with these findings, in our study, no signs of severe weight loss, infection, or wounds were found in any of the treated mice, even in the case of multi‐drug use, implying that oxaliplatin combined with CDK1 inhibitor is well‐tolerated in vivo and thus has a promising clinical application for oxaliplatin‐resistant patients. Nevertheless, inhibitors that specifically target CDK1 have not been translated into clinical use. Currently, the nonspecific CDK1 inhibitors used in clinical trials are multitarget and repress multiple CDKs, such as dinaciclib targeting CDK1/2/5/9, flavopiridol targeting CDK1/2/4/6, AZD5438 targeting CDK1/2/9, and PHA‐793887 targeting CDK1/2/4. Unfortunately, these drugs are either ineffective in the treatment of solid tumors^[^ ^44^ ^]^ or have serious side effects,^[^ ^45^, ^46^ ^]^ limiting their widespread clinical use. This can be explained by the following reasons: i) The efficacy of one drug alone is limited in some cases, and other drugs need to be combined to achieve a therapeutic effect, such as the recent combination of dinaciclib and pembrolizumab.^[^ ^47^ ^]^ ii) There are pros and cons of having multiple drug targets. It affects various signaling pathways under specific circumstances, some of which may be necessary for normal cell survival, resulting in serious side effects. Hence, selective drugs targeting specific proteins need to be developed and utilized. iii) Prior to clinical trials, the recruited patients were not screened based on CDKs expression, which is of vital importance for targeted therapies; patients with low CDKs expression may be insensitive to CDK targeted therapy. Overall, our data provide a solid theoretical basis for the use of CDK1 inhibitors to treat patients with oxaliplatin resistance. Obviously, many factors, including toxicity, dose, tolerance, and patient screening, need to be carefully investigated in preclinical trials before being translated into clinical application.

## 4 Conclusion

In summary, our findings indicate that CDK1 is a previously unappreciated targetable vulnerability in oxaliplatin resistance, and CDK1 inhibitor combined with oxaliplatin represents a promising strategy in the treatment of patients with oxaliplatin‐resistant CRC.

## 5 Experimental Section

### Chemicals

The following chemical reagents were used in this study: Oxaliplatin (#HY‐17371; MedChemExpress, NJ, USA), 5‐Aza‐dC (#A3656; Sigma–Aldrich, MO, USA), Trichostatin A (#V900931; Sigma–Aldrich), chloroquine (#HY‐17589A; MedChemExpress), cycloheximide (#S7418; Selleck Chemicals, TX, USA), MG132 (#M7449; Sigma–Aldrich), RO‐3306 (#S7747; Selleck Chemicals), erastin (#S7242; Selleck Chemicals), RSL3 (#S8155; Selleck Chemicals), ferrostatin‐1 (#S7243; Selleck Chemicals), deferoxamine (#HY‐B1625; MedChemExpress), *N*‐acetyl‐cysteine (#HY‐B0215; MedChemExpress), Z‐VAD‐FMK (#V116; Sigma–Aldrich), necrostatin‐1 (#S8037; Selleck Chemicals), Rosiglitazone (#HY‐17386; MedChemExpress), and liproxstatin‐1 (#HY‐12726; MedChemExpress).

### Cell Lines

HCT8 (RRID: CVCL_2478), HT29 (RRID: CVCL_0320), and HEK293T (RRID: CVCL_HA71) cells were purchased from the American Type Culture Collection (Manassas, VA, USA) and cultured in Dulbecco's modified Eagle's medium (DMEM) supplemented with 10–15% fetal bovine serum. Mycoplasma detection was performed routinely before using the cells. To establish cells resistant to oxaliplatin, HCT8 (RRID: CVCL_2478) and HT29 (RRID: CVCL_0320) cells were exposed to gradually increasing concentrations of oxaliplatin, as previously described.^[^ ^48^ ^]^ Each concentration of oxaliplatin was administered for 2 weeks, for a total duration of approximately 9 months. Stable oxaliplatin‐resistant cells were obtained and cultured in the presence of the final oxaliplatin concentration for at least 6 months. For the functional assays, oxaliplatin was removed for at least 1 week to avoid acute effects.

### CRISPR/Cas9 Screening

HCT8‐OR cells were infected with the pooled GeCKOv2.0 human lentiviral library (MOI = 0.3), followed by puromycin treatment for 1 week. Then, the cells were divided into four parts: two for in vitro screening and two for in vivo screening. For in vitro screening, the mutant HCT8‐OR cells were treated with vehicle or 5 × 10^−6^ m oxaliplatin for 15 days. For in vivo screening, the mutant HCT8‐OR cells were subcutaneously injected into NOD/SCID mice. When tumors reached 150∼200 mm^3^, mice were treated with vehicle or oxaliplatin (5 mg kg^−1^, every 3 days, i.p.) for 15 days. Next, genomic DNA from cells and tumors was purified, followed by sgRNA amplification and deep sequencing. The number of unique reads for each sgRNA was counted, and log_2_‐fold was calculated. After subtracting the median of nontargeting controls, the sgRNA score was obtained. The CRISPR score (CS) was defined as the median score for all sgRNAs targeting a given gene.

### CRC Tissues

A total of 69 fresh tissues (39 cases of oxaliplatin sensitivity and 30 cases of oxaliplatin resistance) were obtained from patients with stage II–III CRC who underwent postoperative oxaliplatin‐based adjuvant chemotherapy (FOLFOX or XELOX regimen). All tissues were stored in liquid nitrogen before use. In addition, 102 paraffin‐embedded tissues from patients with stage II–III CRC were collected to validate the correlation between CDK1 and oxaliplatin resistance. Follow‐up was conducted regularly via telephone or mail. Patients with local relapse were considered oxaliplatin‐resistant. Detailed clinicopathological characteristics are listed in Table S5 (Supporting Information).

### qRT‐PCR

Total RNA was extracted using TRIzol (Invitrogen, CA, USA), followed by reverse transcription into cDNA using the PrimeScript RT reagent Kit (#RR037A; Takara Bio, Otsu, Japan). qPCR was conducted using TB Green Fast qPCR Mix (#RR430A; Takara Bio) according to the manufacturer's instructions. GAPDH was used to normalize the expression of the target genes. The primer sequences used in this study are listed in Table S6 (Supporting Information).

### IHC Staining

Tissues were fixed in 10% formalin, embedded in paraffin, and then cut into slices with a thickness of 4 µm. Antigen retrieval was conducted before incubation with appropriate antibody at 4 °C overnight. The protein signal was visualized using the VECTASTAIN Elite ABC‐HRP Kit (#PK‐6200; Vector Laboratories, CA, USA). Evaluation of IHC staining was conducted by two independent pathologists using the H‐score method as previously described.^[^ ^49^ ^]^ The antibodies used for IHC staining are listed in Table S7 (Supporting Information).

### Immunoblotting (IB) and Co‐IP

Total protein was extracted using the radioimmunoprecipitation assay lysis buffer containing 0.5 m Tris‐HCl, 1.5 m NaCl, 10% NP‐40, 10 × 10^−3^ m EDTA, and 2.5% deoxycholic acid. After centrifugation, the supernatant was used for the standard immunoblotting (IB) assay, as previously described.^[^ ^49^ ^]^ For Co‐IP analysis, the anti‐Flag M2 affinity gel (#A2220; Sigma–Aldrich) or appropriate antibody was added to the supernatant and incubated at 4 °C overnight. After incubation with Pierce Protein A/G Magnetic Beads (#88 802; Thermo Fisher Scientific, MA, USA) for 2 h, the bound proteins were eluted, followed by IB detection using the appropriate antibody. In addition, for in vivo ubiquitylation assay, cells were treated with MG132 for 6 h before lysis, followed by Co‐IP assay. The antibodies used for the IB and Co‐IP assays are listed in Table S7 (Supporting Information).

### Plasmids, Oligonucleotides, and Transfection

For gene overexpression, the full‐length coding sequences of human CDK1, METTL3, YTHDF2, ACSL4, and UBR5 were chemically synthesized and inserted into pcDNA 3.1 (RRID:Addgene_20 407), pFlag‐CMV‐5a (RRID:Addgene_105 933), pCMV‐Myc (RRID:Addgene_73 365), or pCMV‐HA (RRID:Addgene_15 739) vectors as appropriate. HA‐ubiquitin‐WT was obtained from Addgene (Watertown, MA, USA). Site mutation was conducted using the Q5 Site‐Directed Mutagenesis Kit (#E0554S, New England Biolabs, MA, UK) based on the manufacturer's protocols. All constructs were confirmed by Sanger sequencing. All siRNAs were chemically synthesized by Sangon Biotech (Shanghai, China) and validated. The siRNA targeting sequences are listed in Table S6 (Supporting Information). Cell transfection was carried out using Lipofectamine 3000 reagent (Invitrogen).

### Generation of CDK1^−/−^ Cell Lines

Two sgRNAs targeting CDK1 were designed and synthesized (Table S6, Supporting Information), followed by insertion into the lenti‐CRISPR v2 plasmid (RRID:Addgene_87 360). HEK293T (RRID: CVCL_HA71) cells were co‐transfected with lenti‐CRISPR v2 and packaging plasmids (psPAX2 (RRID:Addgene_12 260) and pMD2.G (RRID:Addgene_12 259)). After 72 h, the viral supernatant was collected and used to infect HCT8‐OR and HT29‐OR cells in the presence of polybrene, followed by selection using puromycin for 7 days. Knockout efficiency was verified by Sanger sequencing and IB assays.

### CCK‐8, Colony Formation, and Apoptosis Assays

For CCK‐8 assay, cells were treated with 10 µL CCK‐8 solution (#HY‐K0301; MedChemExpress) at 37 °C for 2 h, the absorbance at 450 nm was measured with a microplate reader. The colony formation assay was conducted by crystal violet staining of cells after 14 days of appropriate treatment. Cell apoptosis was detected using the PE Annexin V Apoptosis Detection Kit (#559 763; BD Biosciences, CA, USA). In brief, cells were treated with 5 µL PE Annexin V and 5 µL 7‐amino‐actinomycin (7‐AAD) in Annexin V binding buffer, followed by analysis of the number of apoptotic cells using a BD Accuri C6 flow cytometer (BD Biosciences). A minimum of 10 000 single cells in each well were used for analysis. The gating strategy is shown in Figure S9 (Supporting Information), and the percentage of apoptotic cells is the sum of the second (late apoptosis) and third (early apoptosis) quadrants.

### m^6^A Detection

Total m^6^A levels were detected using the m^6^A RNA Methylation Quantification Kit (#P‐9005‐48; Epigentek, NY, USA) or by immunofluorescence staining with anti‐m^6^A antibody (RRID:AB_2 924 958). In addition, the enrichment of m^6^A in CDK1 mRNA was detected by the m^6^A‐RNA immunoprecipitation (MeRIP) assay using the Magna MeRIP m^6^A Kit (#17‐10499; Sigma–Aldrich). In brief, total RNA was extracted and chemically fragmented into approximately 100 nt in length, followed by incubation with anti‐m^6^A antibody (RRID:AB_2 924 958). The enriched RNA was eluted and used for qRT‐PCR analysis.

### Luciferase Reporter Assay

Full‐length mature CDK1 mRNA was chemically synthesized and inserted behind the luciferase gene of the pmirGLO vector (RRID:Addgene_117 339, Promega, WI, USA), followed by transfection into cells using Lipofectamine 3000. Cell lysates were collected after 48 h of transfection for luciferase activity detection using the dual‐luciferase reporter assay system (Promega). The renilla luciferase was used as the reference control.

### RNA Immunoprecipitation Assay

Cell lysate was collected and incubated with appropriate antibody at 4 °C overnight. After incubation with Pierce Protein A/G Magnetic Beads (Thermo Fisher Scientific) at 4 °C for 3 h, the immunocomplexes were eluted at 65 °C for 10 min in the presence of the elution buffer. Then, proteinase K was added to avoid protein interference, and RNA was purified using TRIzol solution, followed by qRT‐PCR analysis.

### Ni‐NTA Pull‐down Assay

Human recombinant ACSL4 (#TP305356; OriGene, Nanjing, China) and His‐CDK1 (#ab187447; Abcam, Cambridge, UK) were added to the binding buffer (150 × 10^−3^ m NaCl, 100 × 10^−3^ m NaF, 50 × 10^−3^ m Tris‐HCl, pH 7.6, 0.5% NP‐40, 1 × 10^−3^ m PMSF) and incubated with rotation at 4 °C for 2 h. Then, the Ni‐NTA agarose (#30 230; QIAGEN, Dusseldorf, Germany) was added and incubated at 4 °C for another 30 min, followed by washing and elution using buffer with increasing concentrations of imidazole. The eluted proteins were collected and mixed with sodium dodecyl sulfate‐polyacrylamide gel electrophoresis loading buffer for IB analysis.

### In Vitro Phosphorylation Assay

HEK293T (RRID: CVCL_HA71) cells were transfected with Flag‐ACSL4‐WT or Flag‐ACSL4‐S447A vectors. After 72 h, the proteins were immunoprecipitated by anti‐Flag M2 affinity gel (Sigma–Aldrich). Then, the purified Flag‐ACSL4‐WT or Flag‐ACSL4‐S447A was incubated with CDK1/Cyclin B (#14‐450; Sigma‐Aldrich) in phosphorylation buffer (50 × 10^−3^ m Tris‐HCl, pH 7.5, 100 × 10^−3^ m KCl, 5 × 10^−3^ m MgCl_2_, 1 × 10^−3^ m Na_3_VO_4_, 50 × 10^−3^ m DTT, 5% glycerol, 50 × 10^−3^ m ATP) at 37 °C for 1 h. The reaction was terminated by adding SDS‐PAGE loading buffer, followed by IB analysis with the appropriate antibody.

### Generation of Antibodies Against p‐ACSL4 S447

p‐ACSL4 S447 polyclonal antibody was produced by Genscript (NJ, USA) from rabbits. The amino acid sequence for immunization was GAPLSPQTHRFMNVC, where S denotes the phosphorylated residue in the synthetic peptide.

### In Vitro Ubiquitination Assay

The assay was carried out by using the in vitro ubiquitinylation kit (#BML‐UW9920; Enzo Life Sciences, Raamsdonksveer, NL) according to the manufacturer's instructions. In brief, 1 × 10^−6^ m ACSL4 and 200 × 10^−9^ m UBR5 were incubated with 1×ubiquitination buffer, 20 U mL^−1^ inorganic pyrophophatase, 1 × 10^−3^ m dithiothreitol, 5 × 10^−3^ m Mg‐ATP, 2.5 × 10^−6^ m ubiquitin, 100 × 10^−9^ m E1 enzymes, and 2.5 × 10^−6^ m various E2 enzymes in a total reaction volume of 50 µL at 37 °C for 1 h. Then, the reaction was quenched by the nonreducing gel loading buffer, followed by IB analysis.

### Assessment of Cell Death and Lipid Peroxidation

Cell death was assessed via propidium iodide (PI) staining, followed by flow cytometric analysis. PI‐positive cells were considered dead. Lipid peroxidation was tested using C11‐BODIPY 581/591 staining. In brief, cells were incubated with 5 × 10^−6^ m BODIPY 581/591 dye (#D3861; Thermo Fisher Scientific) for 30 min at 37 °C, followed by flow cytometric analysis. The ratio of FITC (oxidized form) to PE (reduced form) fluorescence intensity was used to measure lipid peroxidation. A minimum of 10 000 cells were analyzed per condition. In addition, the Fe^2+^ content was tested by the Iron Assay Kit (#ab83366; Abcam) according to the manufacturer's manual, followed by measurement of the absorbance at 593 nm with a microplate reader.

### Animal Study

To establish CDX models, HCT8‐OR or HT29‐OR cells with or without CDK1 knockout were subcutaneously injected into NOD/SCID mice, which were randomly divided into five groups and housed under specific pathogen‐free conditions. When tumors reached 150–200 mm^3^, mice were treated with oxaliplatin (5 mg kg^−1^, every 3 days, i.p.) alone, or in combination with RO‐3306 (4 mg kg^−1^ every 2 days, oral gavage) and liproxstatin‐1 (10 mg kg^−1^ once daily, i.p.). Tumor volume (mm^3^) was recorded every 5 days using the formula: Volume = length × width^2^ × 1/2. All mice were euthanized at the end of the experiment, tumor weights were recorded, and tissues were collected for IHC staining.

To establish the PDX models, fresh clinical tissues were obtained from patients with surgically resected CRC who had undergone three cycles of oxaliplatin‐based neoadjuvant chemotherapy (Table S4, Supporting Information). Tumor response was evaluated using the Response Evaluation Criteria in Solid Tumors version 1.1. Specifically, complete or partial response was considered oxaliplatin sensitivity, while stable or progressive disease was considered oxaliplatin resistance. The tissues were placed in cold DMEM, immediately diced into 2–3 mm^3^ pieces, and subsequently implanted into the flanks of NOD/SCID mice. When tumors reached about 1–1.5 cm^3^, they were excised for cohort expansion. Drug administration began after tumors reached approximately 150–200 mm^3^ (*n* = 5 per group), followed by evaluation of tumor size and IHC staining, as shown above.

### Study Approval

Clinical CRC tissues were obtained from patients who provided written informed consent prior to their inclusion in the study. This study was approved by the Ethics Committee of Sun Yat‐sen University Cancer Center (approval No: G2021‐088‐01). All animal experiments were conducted in accordance with the guidelines of the Animal Care and Use Committee of Sun Yat‐sen University Cancer Center (approval No: SYSU‐IACUC‐2021‐000653).

### Statistical Analysis

Data are presented as the mean ± standard deviation (SD). Differences between two groups were analyzed using the two‐tailed unpaired Student's *t*‐test (measurement data) or chi‐square test (enumeration data), whereas those among three or more groups were analyzed using one‐ or two‐way analysis of variance coupled with a post‐hoc test. Kaplan–Meier method was used to plot the survival curves of patients with CRC and their differences were compared using the log‐rank test. Correlation analysis was performed using the Spearman rank correlation coefficient. The statistical analysis was conducted by using GraphPad Prism 7.0 (GraphPad Software Inc., CA, USA). Statistical significance was set at *p* < 0.05.

## Conflict of Interest

The authors declare no conflict of interest.

## Supporting information

Supporting Information

Supplemental Table 1

## Data Availability

The data that support the findings of this study are available in the supplementary material of this article.
