# Supplementary material for: Inhibition of CDK1 Overcomes Oxaliplatin Resistance by Regulating ACSL4‐mediated Ferroptosis in Colorectal Cancer
Source: Adv Sci (Weinh). 2023 Jul 10;10(25):2301088. doi: 10.1002/advs.202301088 (PMC10477855; doi:10.1002/advs.202301088)
Supplement: Supplementary file 1 — Supporting Information [file ADVS-10-2301088-s001.pdf]

ADVANCED SCIENCE
Open Access

Supporting Information

for *Adv. Sci.*, DOI 10.1002/advs.202301088

Inhibition of CDK1 Overcomes Oxaliplatin Resistance by Regulating ACSL4-mediated Ferroptosis in Colorectal Cancer

Kaixuan Zeng, Weihao Li, Yue Wang, Zifei Zhang, Linjie Zhang, Weili Zhang, Yue Xing* and Chi Zhou**

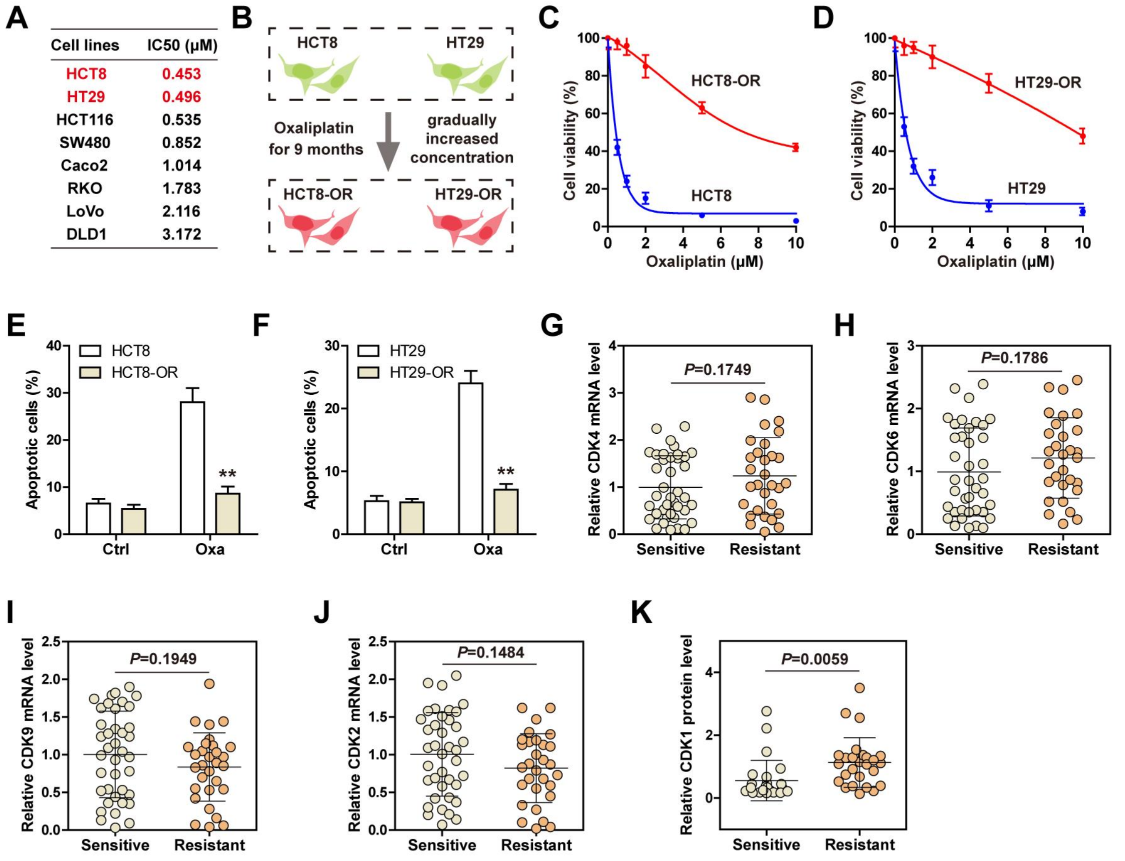

Figure S1. Construction of oxaliplatin-resistant CRC cell lines. A. The IC50 values of oxaliplatin in the indicated CRC cell lines. B. The cartoon showing the process of constructing oxaliplatin-resistant HCT8-OR and HT29-OR cell lines. C, D. CCK-8 assay testing cell viability in the indicated cells following treatment with different concentrations of oxaliplatin. E, F. Flow cytometry analyzing the death of HCT8-OR and HT29-OR cells after 1μM oxaliplatin treatment for 48h. G-J. qRT-PCR analysis of CDK2, CDK4, CDK6, and CDK9 mRNA levels in oxaliplatin-sensitive (n=39) and -resistant (n=30) CRC tissues. K. Image J quantifying the protein level of CDK1 in Figure 1D. The data are shown as the mean ± SD (n=3). $^{**}P<0.01$, by 2-way ANOVA test (E, F) or 2-tailed unpaired Student's t test (G-K). OR, oxaliplatin resistance; Oxa, oxaliplatin.

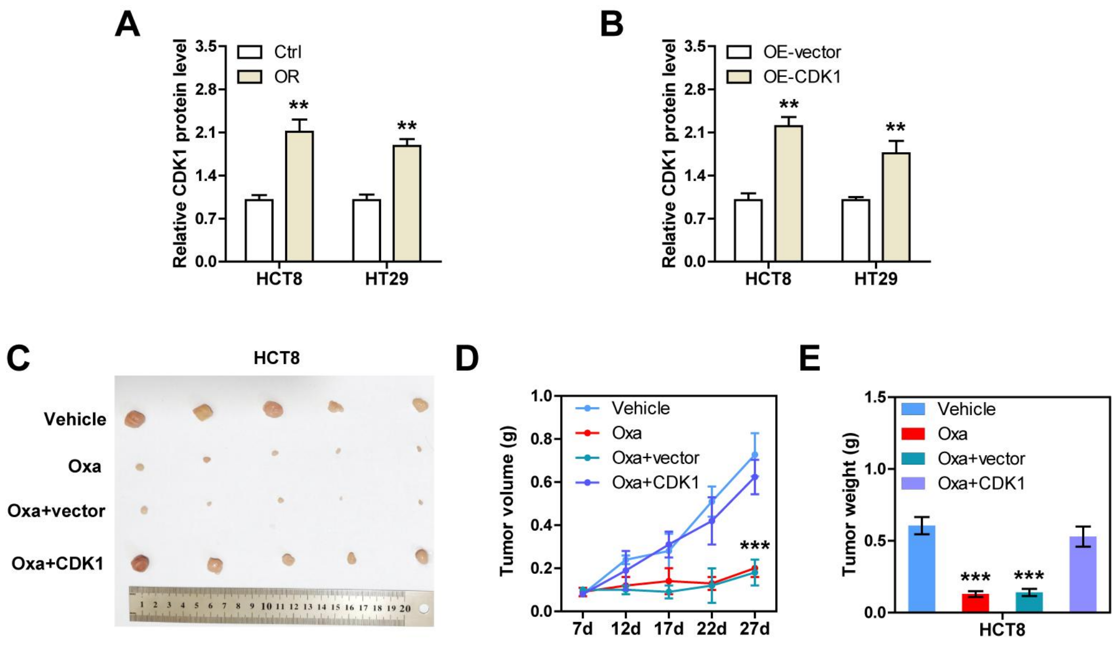

Figure S2. CDK1 is linked to oxaliplatin resistance. A. Image J quantifying the protein level of CDK1 in Figure 2B (n=3). B. Image J quantifying the protein level of CDK1 in Figure 2H (n=3). C-E. The image, volume and weight of tumor generated by HCT8 cells in vehicle, Oxa (5mg/kg), Oxa+vector and Oxa+CDK1 groups (n=5). The data are shown as the mean ± SD. $^{**}P$<0.01, $^{***}P$<0.001, 2-tailed unpaired Student's t test (A, B), 2-way ANOVA (D) or 1-way ANOVA (E) followed by Tukey's post hoc test. OR, oxaliplatin resistance; Oxa, oxaliplatin.

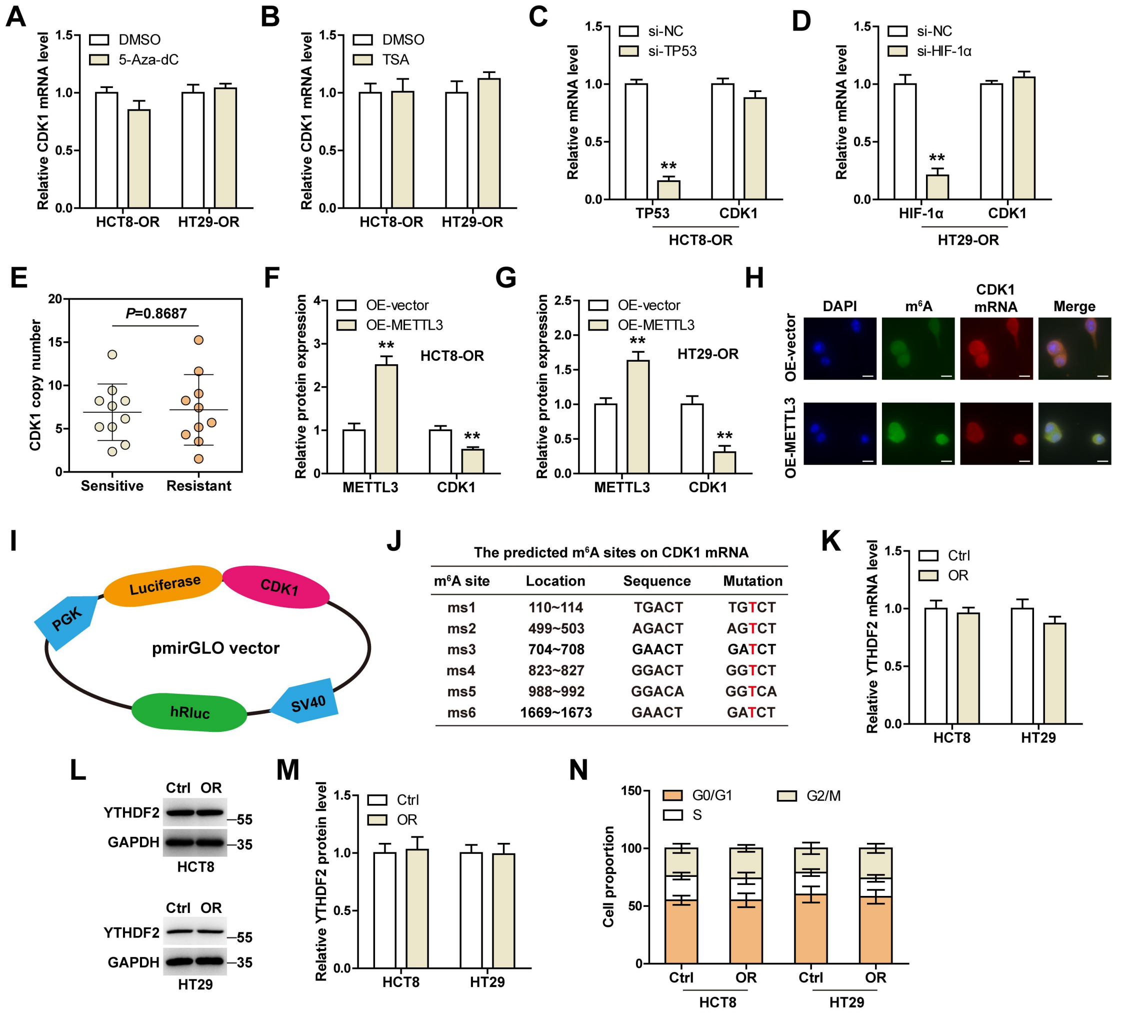

Figure S3. Detection of the cause of CDK1 upregulation. A, B. qRT-PCR analysis of CDK1 mRNA levels in HCT8-OR and HT29-OR cells treated with 20μM 5-Aza-dC or 100nM Trichostatin A for 24h. C, D. qRT-PCR analysis of the indicated gene expression in HCT8-OR and HT29-OR cells transfected with si-TP53 or si-HIF-1α. E. Analysis of the copy number of CDK1 in oxaliplatin-sensitive (n=10) and -resistant (n=10) CRC tissues. F, G. Image J quantifying the protein levels of METTL3 and CDK1 in Figure 3E. H. Immunofluorescence testing the co-localization of m^6A and CDK1 mRNA in HCT8-OR cells with or without METTL3 overexpression. Scale bars, 20μm. I. The schematic diagram of constructing the pmirGLO-CDK1 vector. J. Six m^6A sites on CDK1 mRNA were predicted by SRAMP online tool. K. qRT-PCR analysis of YTHDF2 mRNA levels in the indicated cell lines. L, M. IB testing YTHDF2 protein levels in the indicated cell lines. N. PI staining testing the distribution of cell cycle in the indicated cells. The data are shown as the mean ± SD. $^{**}P<0.01$, 2-tailed unpaired Student's t test (A-D, F, G, K, M, N). TSA, Trichostatin A; ms, m^6A site; Ctrl, control; OR, oxaliplatin resistance. The statistics of all panels were determined by 2-tailed unpaired Student's t test.

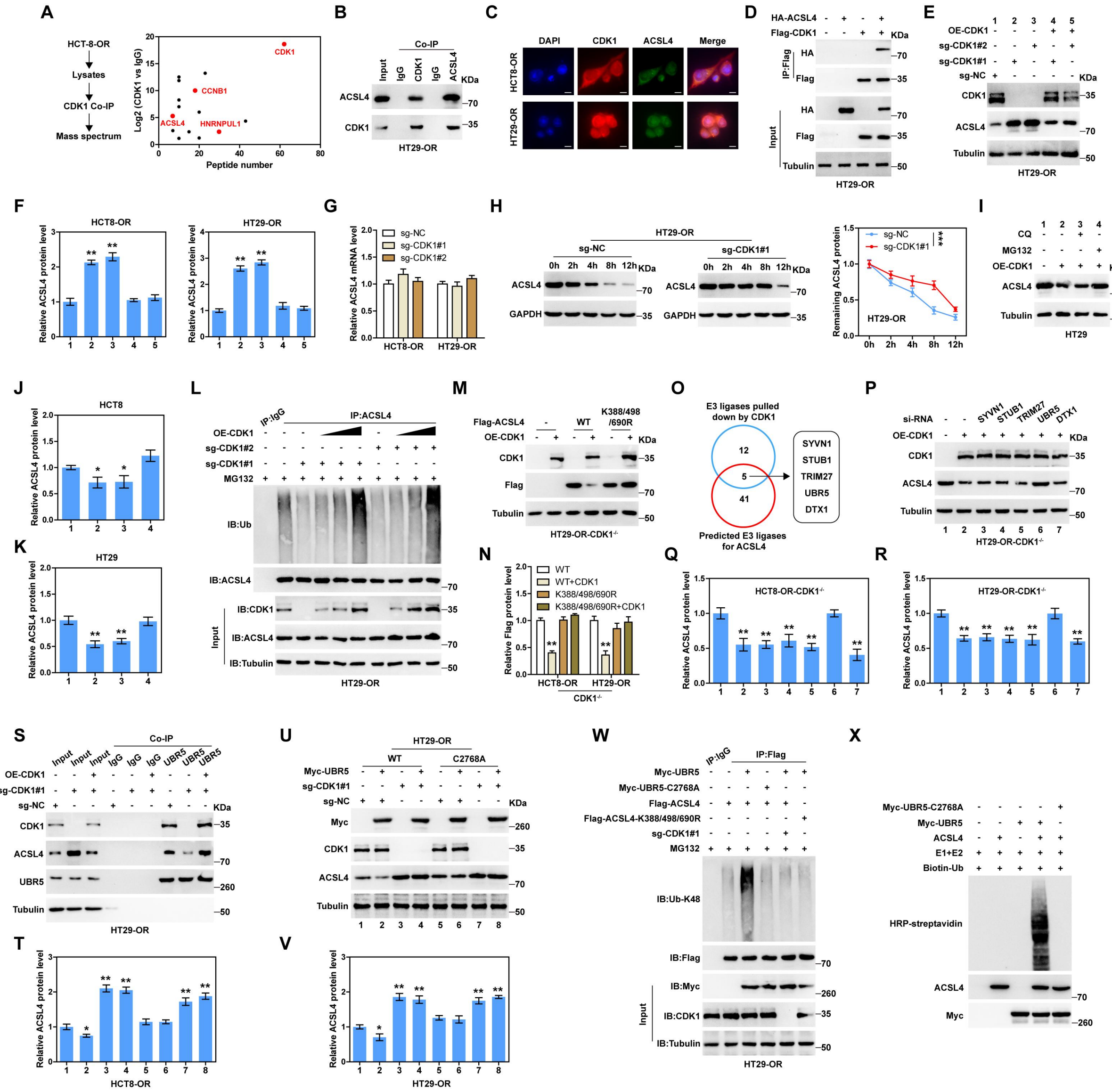

Figure S4. CDK1 interacts with and promotes ACSL4 protein degradation. A. IP assay using anti-CDK1 antibody in HCT8-OR cells, followed by mass spectrometry. B. IP assay testing the endogenous interaction between CDK1 and ACSL4. C. Immunofluorescence testing the co-localization of CDK1 and ACSL4 proteins in HCT8-OR and HT29-OR cells. Scale bars, 20μm. D. HT29-OR cells transfected with the labeled plasmids for 48h, followed by IP coupled with IB assays using the indicated antibodies. E, F. IB assay testing the expression of CDK1 and ACSL4 proteins in CDK1 knockout cells, followed by quantification by Image J. G. qRT-PCR analysis of ACSL4 mRNA levels in CDK1 knockout cells. H. IB assay testing ACSL4 expression in CDK1 knockout HT29-OR cells treated with 100 μg/mL cycloheximide for the indicated time. I-K. IB assay testing ACSL4 expression in CDK1-overexpressing cells treated with 5 μM MG132 or 10 μM chloroquine for 12h, followed by quantification by Image J. L. IP assay using anti-ACSL4 antibody, followed by IB assay with the indicated antibodies in HT29-OR cells treated with 20μM MG132 for 6h. M, N. IB assays detecting the indicated protein levels in CDK1

knockout cells transfected with the indicated vectors. O. Five E3 ligases predicted to be interacted with CDK1 and ACSL4, simultaneously. P-R. IB assay testing the ACSL4 protein levels in CDK1 knockout cells transfected with the indicated vectors or siRNAs, followed by quantification by Image J. S. IP assay using anti-UBR5 antibody, followed by IB assay with the indicated antibodies in HT29-OR cells. T-V. IB assay detecting the ACSL4 protein levels in CDK1 knockout HT29-OR cells transfected with wild-type or mutant UBR5 plasmid, followed by quantification by Image J. W. HT29-OR cells were transfected with the indicated vectors, followed by IP coupled with IB assays using the indicated antibodies. X. The *in vitro* ubiquitination assay testing the direct ubiquitination of UBR5 on ACSL4. The data are shown as the mean ± SD. $^{**}P<0.01$, $^{***}P<0.001$, by 1-way ANOVA (G, N) or 2-way ANOVA followed by Tukey's post hoc test (H). NC, negative control; OE, overexpressed; WT, wild-type; CQ, chloroquine.

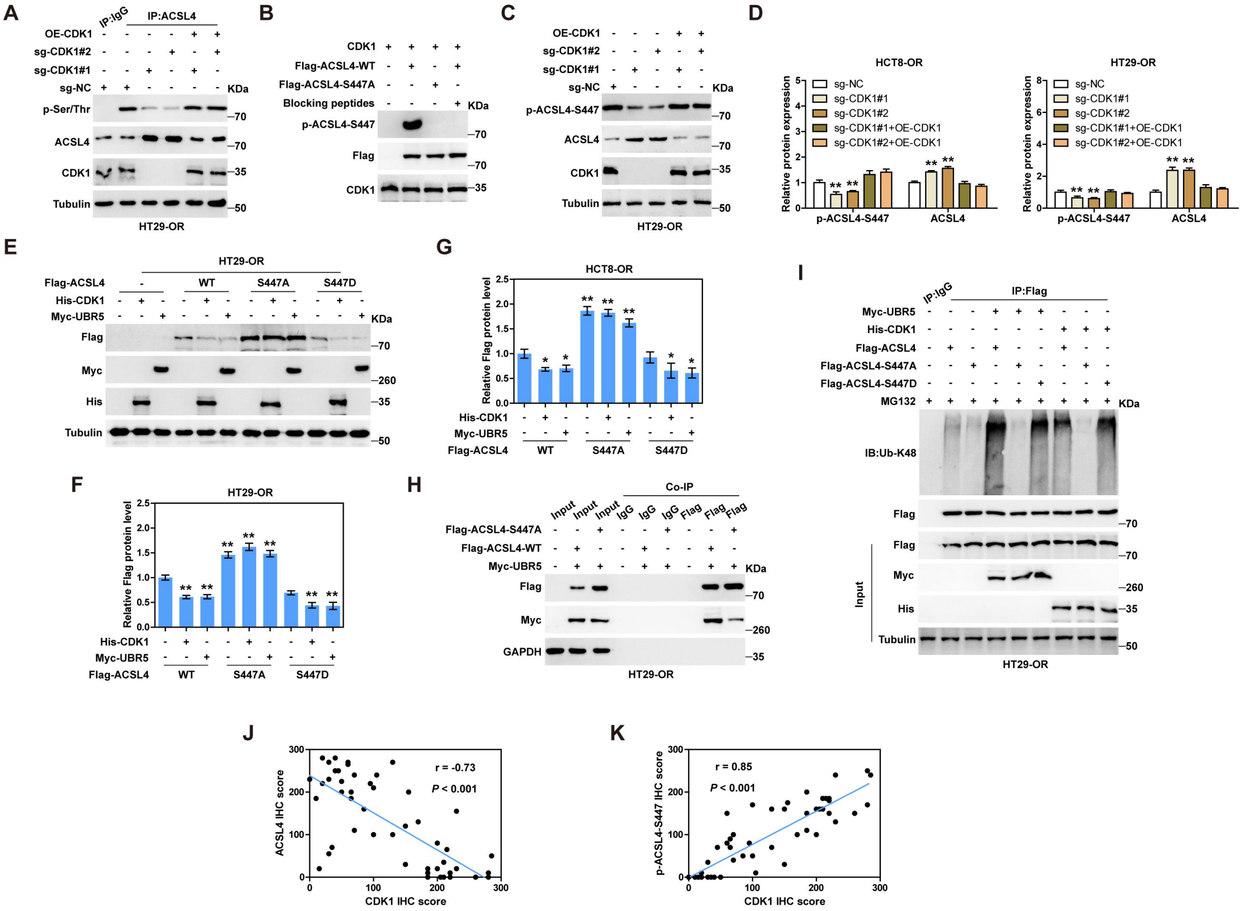

Figure S5. CDK1 phosphorylates ACSL4 protein at S447. A. IP coupled with IB assays using the indicated antibodies testing the effect of CDK1 on ACSL4 phosphorylation. B. *In vitro* phosphorylation assay using the purified proteins verifying the effectiveness of anti-p-ACSL4 S447 antibody. C, D. IB assay using the indicated antibodies in cells transfected with the indicated vectors, followed by quantification by Image J. E-G. IB assay using the indicated antibodies in cells transfected with the indicated vectors, followed by quantification by Image J. H, I. IP coupled with IB assays using the indicated antibodies in HT29-OR cells transfected with the indicated vectors. J, K. The correlation between CDK1 and ACSL4 or p-ACSL4 S447 in CRC tissues. Statistical analysis was conducted using Spearman rank correlation coefficient. The data are shown as the mean ± SD. $^{*}P<0.05$, $^{**}P<0.01$, by 1-way ANOVA (D, G, F) followed by Tukey's post hoc test. NC, negative control; OE, overexpressed; WT, wild-type.

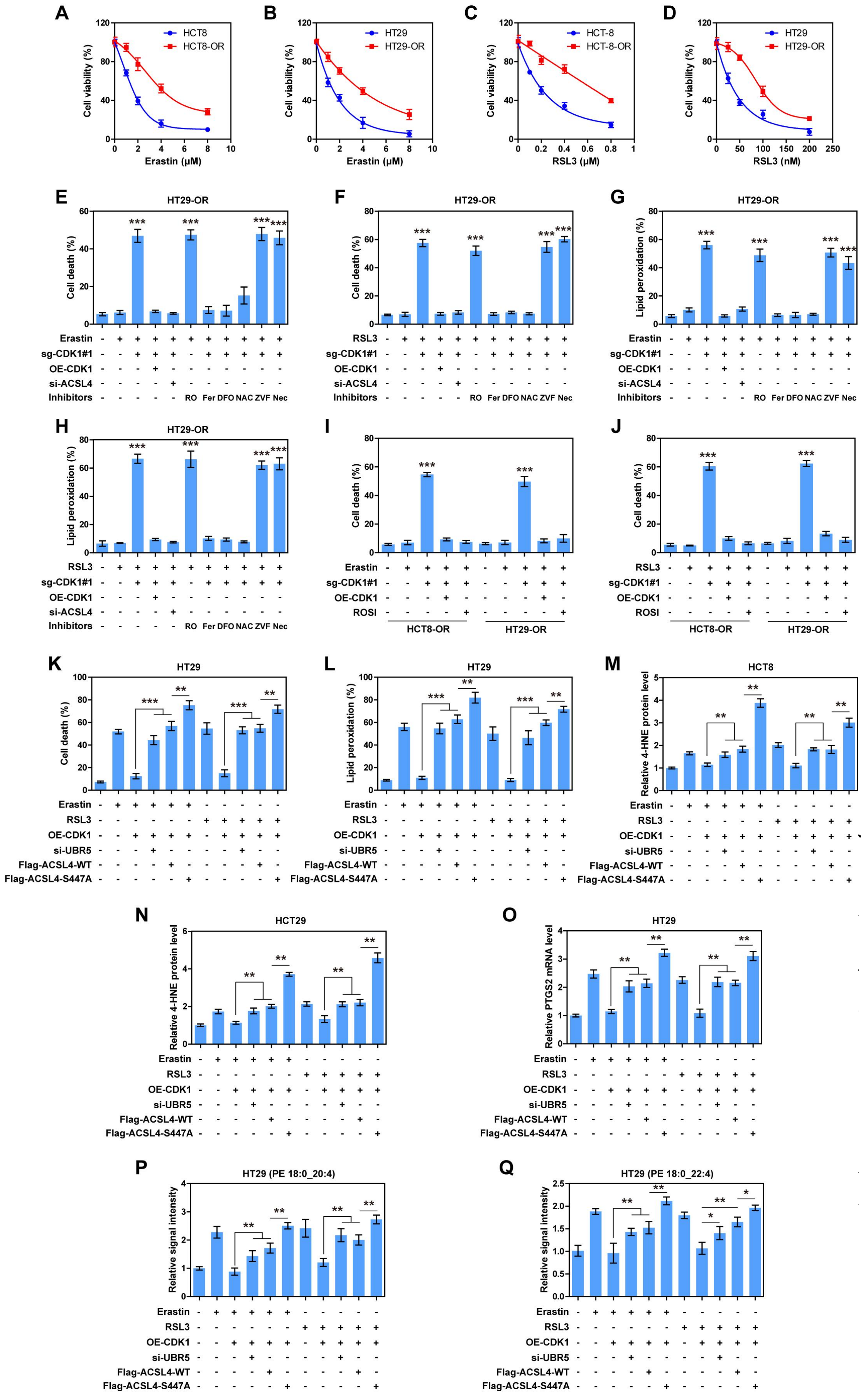
A
Cell viability (%)
Erastin (μM)
HCT8
HCT8-OR
B
Cell viability (%)
Erastin (μM)
HT29
HT29-OR
C
Cell viability (%)
RSL3 (μM)
HCT-8
HCT-8-OR
D
Cell viability (%)
RSL3 (nM)
HT29
HT29-OR
E
HT29-OR
Cell death (%)
Erastin
sg-CDK1#1
OE-CDK1
si-ACSL4
Inhibitors
RO Fer DFO NAC ZVF Nec
F
HT29-OR
Cell death (%)
RSL3
sg-CDK1#1
OE-CDK1
si-ACSL4
Inhibitors
RO Fer DFO NAC ZVF Nec
G
HT29-OR
Lipid peroxidation (%)
Erastin
sg-CDK1#1
OE-CDK1
si-ACSL4
Inhibitors
RO Fer DFO NAC ZVF Nec
H
HT29-OR
Lipid peroxidation (%)
RSL3
sg-CDK1#1
OE-CDK1
si-ACSL4
Inhibitors
RO Fer DFO NAC ZVF Nec
I
Cell death (%)
Erastin
sg-CDK1#1
OE-CDK1
ROSI
HCT8-OR
HT29-OR
J
Cell death (%)
RSL3
sg-CDK1#1
OE-CDK1
ROSI
HCT8-OR
HT29-OR
K
HT29
Cell death (%)
Erastin
RSL3
OE-CDK1
si-UBR5
Flag-ACSL4-WT
Flag-ACSL4-S447A
L
HT29
Lipid peroxidation (%)
Erastin
RSL3
OE-CDK1
si-UBR5
Flag-ACSL4-WT
Flag-ACSL4-S447A
M
HCT8
Relative 4-HNE protein level
Erastin
RSL3
OE-CDK1
si-UBR5
Flag-ACSL4-WT
Flag-ACSL4-S447A
N
HCT29
Relative 4-HNE protein level
Erastin
RSL3
OE-CDK1
si-UBR5
Flag-ACSL4-WT
Flag-ACSL4-S447A
O
HT29
Relative PTGS2 mRNA level
Erastin
RSL3
OE-CDK1
si-UBR5
Flag-ACSL4-WT
Flag-ACSL4-S447A
P
HT29 (PE 18:0_20:4)
Relative signal intensity
Erastin
RSL3
OE-CDK1
si-UBR5
Flag-ACSL4-WT
Flag-ACSL4-S447A
Q
HT29 (PE 18:0_22:4)
Relative signal intensity
Erastin
RSL3
OE-CDK1
si-UBR5
Flag-ACSL4-WT
Flag-ACSL4-S447A

Figure S6. CDK1 represses ACSL4-mediated ferroptosis. A-D. CCK-8 assay testing cell viability after treatment with different concentrations of erastin or RSL3. E-J. The indicated vectors or siRNAs were transfected into cells treated with 1μM erastin, 100nM RSL3, 2μM RO-3306, 5μM ferrostatin-1, 5μM deferoxamine, 5mM N-acetyl-cysteine, 10μM Z-VAD-FMK, 2μM necrostatin-1, or 5μM rosiglitazone for 20h, followed by detection of cell death and lipid peroxidation. K, L. The indicated vectors or siRNAs were transfected into HT29 cells treated with 2μM erastin, 200nM RSL3 for 20h, followed by detection of cell death and lipid peroxidation. M, N. Image J quantifying the protein levels of 4-HNE in Figure 6G, H. O. qRT-PCR analysis of PTGS2 mRNA levels in HT29 cells transfected with the indicated vectors or siRNAs and treated with 2μM erastin or 200nM RSL3 for 20h. P, Q. Mass spectrometric analysis of the signal intensities of PE 18:0_20:4 and PE 18:0_22:4 in HT29cells transfected with the indicated vectors or siRNAs and treated with 2μM erastin or 200nM RSL3 for 20h. The data are shown as the mean ± SD. $^{*}P<0.05$, $^{**}P<0.01$, $^{***}P<0.001$, by 1-way ANOVA followed by Tukey's post hoc test (E-Q). OE, overexpressed; WT, wild-type. RO, RO-3306; Fer, ferrostatin-1; DFO, deferoxamine; NAC, N-acetyl-cysteine; ZVF, Z-VAD-FMK; Nec, necrostatin-1; ROSI, rosiglitazone.

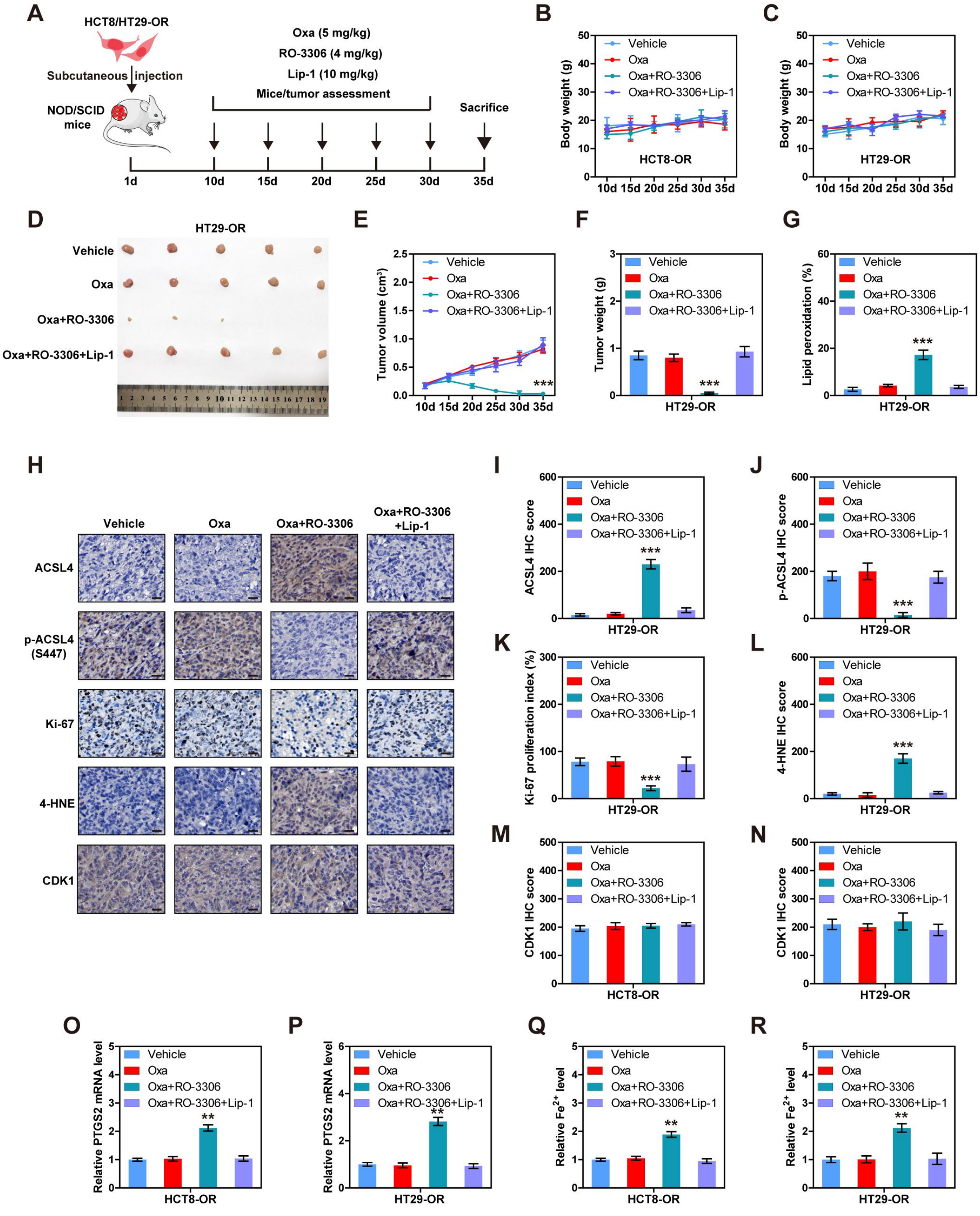

Figure S7. CDK1 inhibitor restores the sensitivity of oxaliplatin in CDX models. A. The diagrammatic sketch of constructing CDX models, followed by treatment with the indicated drugs. B, C. The weight of the mice in the indicated four groups. D-F. The volume, weight and image of tumor generated by HT29-OR injection in the indicated groups (n=5). G. Flow cytometry analyzing lipid peroxidation levels of HT29-OR tumor cells isolated from mice in the indicated groups. H-N. IHC staining of ACSL4, p-ACSL4-S447, Ki-67, 4-HNE and CDK1 in tumor tissues generated by HT29-OR injection in the indicated groups. Scale bars, 50μm. O, P. qRT-PCR analysis of PTGS2 mRNA expression in the indicated groups. Q, R. The ferrous content in tumor tissues in the indicated groups. $^{**}P<0.01$, $^{***}P<0.001$, by 1-way

ANOVA (F, G, I-R) or 2-way ANOVA followed by Tukey's post hoc test (B, C, E). Oxa, oxaliplatin; Lip-1, liproxstatin-1.

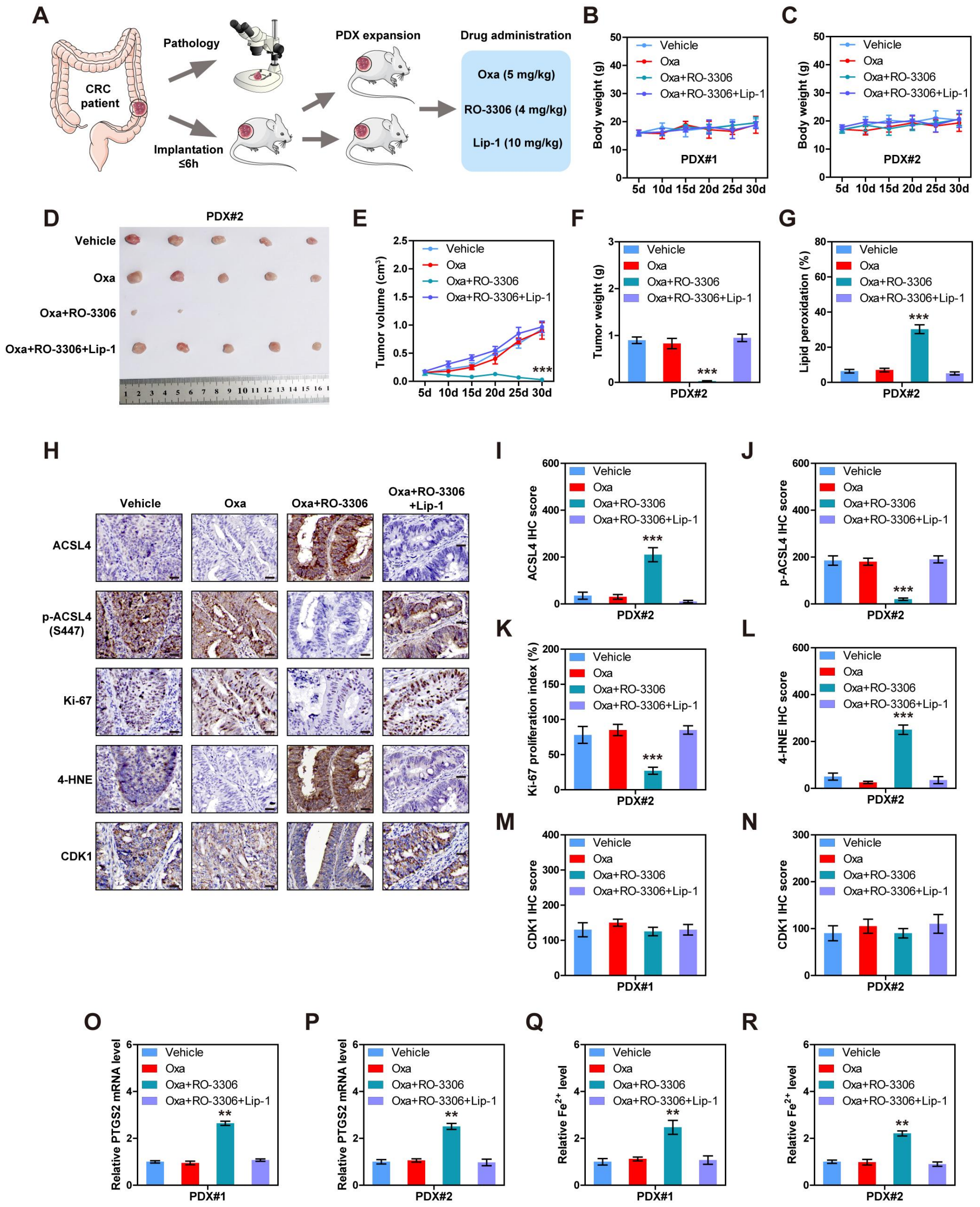

Figure S8. CDK1 inhibitor restores the sensitivity of oxaliplatin in PDX models. A. The diagrammatic sketch of constructing PDX models, followed by treatment with the indicated drugs. B, C. The weight of the mice in PDX#1 and PDX#2 groups. D-F. The image, volume and weight of tumor generated by xenotransplantation of clinical CRC tissues (PDX#2) in the indicated four groups (n=5). G. Flow cytometry detecting lipid peroxidation levels of tumor cells isolated from mice in PDX#2 models.

H-N. IHC staining of CDK1, ACSL4, p-ACSL4-S447, Ki-67 and 4-HNE in tumor tissues generated by xenotransplantation of clinical CRC tissues in the indicated groups. Scale bars, 50 μm. O, P. qRT-PCR analysis of PTGS2 mRNA expression in the indicated groups. Q, R. The ferrous content in tumor tissues in the indicated groups. $^{**}P<0.01$, $^{***}P<0.001$, by 1-way ANOVA test (F, G, I-R) or 2-way ANOVA test (B, C, E). Oxa, oxaliplatin; Lip-1, liproxstatin-1.

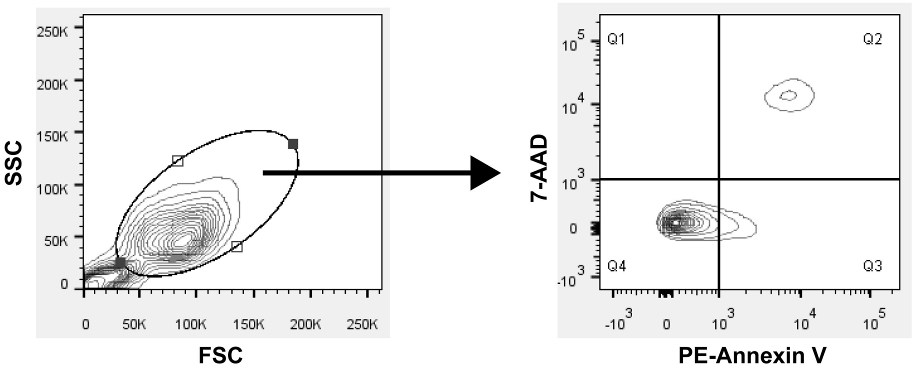

Figure S9. Gating strategy for apoptosis. Using the FSC/SSC gating to remove the debris or attached cells, followed by detection of apoptosis using PE and PerCP/Cy5.5 channels for Annexin V and 7-AAD, respectively.
